# Distribution, community structure and assembly patterns of phytoplankton in the northern South China Sea

**DOI:** 10.3389/fmicb.2024.1450706

**Published:** 2024-07-31

**Authors:** Jian Zou, Yayuan Xiao, Peng Wu, Teng Wang, Lin Lin, Yu Liu, Yong Liu, Chunhou Li

**Affiliations:** ^1^Key Laboratory of South China Sea Fishery Resources Exploitation and Utilization, Ministry of Agriculture and Rural Affairs, South China Sea Fisheries Research Institute, Chinese Academy of Fishery Science, Guangzhou, China; ^2^Scientific Observation and Research Station of Pearl River Estuary Ecosystem of Guangdong Province, Guangzhou, China; ^3^Observation and Research Station of Xisha Island Reef Fishery Ecosystem of Hainan Province, Key Laboratory of Efficient Utilization and Processing of Marine Fishery Resources of Hainan Province, Sanya Tropical Fisheries Research Institute, Sanya, China

**Keywords:** phytoplankton, cyanobacteria, community structure, assembly patterns, cold seep

## Abstract

A cruise was conducted in the summer of 2023 from the Pearl River Estuary (PRE) to the adjacent waters of the Xisha Islands in the northern South China Sea (NSCS) to investigate the distribution, community structure, and assembly patterns of eukaryotic and prokaryotic phytoplankton using high-throughput sequencing (HTS) and microscopic observation. Dinophyta were the most abundant phylum in the eukaryotic phytoplankton community based on HTS, accounting for 92.17% of the total amplicon sequence variants (ASVs). Syndiniales was the most abundant order among eukaryotic phytoplankton, whereas *Prochlorococcus* was the most abundant genus within cyanobacteria. The alpha diversity showed the lowest values in the PRE area and decreased gradually with depth, while cyanobacteria exhibited higher alpha diversity indices in the PRE and at depths ranging from 75 m to 750 m. The morphological results were different from the data based on HTS. Diatoms (37 species) dominated the phytoplankton community, with an average abundance of 3.01 × 10^4^ cells L^−1^, but only six species of dinoflagellate were observed. Spearman correlation analysis and redundancy analysis (RDA) showed that the distribution and community structure of phytoplankton were largely influenced by geographical location and environmental parameters in the NSCS. The neutral community model (NCM) and null model indicated that deterministic processes played a significant role in the assembly of eukaryotic phytoplankton, with heterogeneous selection and homogeneous selection accounting for 47.27 and 29.95%, respectively. However, stochastic processes (over 60%) dominated the assembly of cyanobacteria and undominated processes accounted for 63.44%. In summary, the formation of eukaryotic phytoplankton was mainly influenced by environmental factors and geographic location, but the assembly of cyanobacteria was shaped by both stochastic processes, which accounted for over 60%, and environmental selection in the NSCS.

## 1 Introduction

Marine phytoplankton, as a primary producer, contributes approximately 50% of global primary productivity despite accounting for less than 1% of biomass (Field et al., 1998). These autotrophic organisms are the basis of the food web and play a critical role in energy flow and substance circulation (Wang et al., 2022). The northern South China Sea (NSCS) is located on the tropical and subtropical continental slope and has been significantly influenced by human activities (Huang et al., 2018). The region includes the Pearl River Estuary (PRE), which is the third largest river in China and exhibits a runoff of 3,260 × 10^8^ m^3^/year, resulting in the eutrophication of waters around the PRE (Huang et al., 2003). The abundance and community structure of phytoplankton are sensitive to changes in nutrient concentrations. Previous studies have shown significant correlations between nutrients and microalgae (Cui et al., 2018; Zou et al., 2022). Even, one study reported that there were 87 occurrences of harmful algal blooms (HABs) in the PRE between 1980 and 2016 (Li et al., 2019). Cold seeps are areas where low-temperature fluids, such as methane, seep into the water from the seabed. These fluids can serve as energy sources for chemoheterotrophs, including shellfish and microorganisms (Pile and Young, 1999; Zhao Y. et al., 2020). Recently, there have been occurrences of phytoplankton in the deep cold seeps, although some studies consider these microalgae to be dead or resting cells sinking from the euphotic layer (Li et al., 2020). However, some heterotrophic phytoplankton such as *Synechococcus* (Cyanophyta) and Syndiniales (Dinophyta) can obtain sufficient carbon from methane and survive in cold seeps. These microalgae are one of the nitrogen sources for symbiotic bivalves (Pile and Young, 1999; Diao et al., 2023). It is crucial to investigate the composition and structure of understudied phytoplankton communities in deep-sea cold spring ecosystems.

The composition of the phytoplankton community plays an important role in the dynamics of the food web and fisheries production (Field et al., 1998) and is also an effective tool for assessing the toxins produced by harmful microalgae (Dufrêne and Legendre, 1997). The methods used in phytoplankton identification were mostly based on morphology in previous studies, but the identified species (cyanobacteria especially) were limited and it could not accurately assess the compositions of microalgae. The development of molecular technology, especially the use of high-throughput sequencing (HTS) technologies, has greatly enriched the diversity of phytoplankton communities, particularly in regard to pico-size phytoplankton, such as cyanobacteria (Kang et al., 2022). It can be achieved that accurate assessment of the composition and abundance in eukaryotic and prokaryotic phytoplankton communities based on the combination of morphological identification and HTS (Peng et al., 2023).

Phytoplankton are widely distributed in marine euphotic waters from 0 m to 200 m, but most research has focused on surface waters from 0 m to 5 m, with little attention given to the distribution and composition of deeper species (Zhang W. et al., 2022; Peng et al., 2023). Some studies on shallow waters have found that depth is an important factor affecting the structure of the phytoplankton community (Jäger et al., 2008). The effects of hydrological conditions such as temperature and salinity on the composition and abundance of phytoplankton have been reported in many studies in shallow or coastal waters (Cui et al., 2018; Peng et al., 2023). Nutrients are indeed key factors that drive the dynamic variations of phytoplankton. Recently, one study found that the community structure of phytoplankton was significantly correlated with dissolved inorganic phosphorus (DIP) and dissolved inorganic nitrogen (DIN) in shallow waters of the Xisha Islands (Peng et al., 2023). In addition, some studies summarized previous studies and found nutrients played an important role in the outbreaks of HABs (Lu et al., 2022). However, the scientific understanding of how hydrological conditions and human activities impact the diversity and distribution patterns of phytoplankton is still limited so far, especially in offshore deep waters (Xu et al., 2024). The mechanism of community assembly has long been a question in aquatic ecology, and it is of great significance for understanding the succession of biological communities (Dini-Andreote et al., 2015; Zhao L. et al., 2020). Deterministic processes (i.e., environmental selection) are often considered the primary factors driving community change (Jiao et al., 2020). However, stochastic processes, including mortality, birth, speciation, and ecological drift, may be the predominant processes driving the formation of biological communities. These stochastic processes can lead to random fluctuations and successions in biological communities, thus deepening our understanding of the mechanisms of community formation (Dini-Andreote et al., 2015;Chen et al., 2019; Guo et al., 2023). Recently, the neutral community model (NCM), originating from Sloan et al. (2006) and studying microbial assembly patterns, has gradually been applied to protist communities. This reveals that stochastic processes also play an important role in the assembly of protists, including phytoplankton (Chen et al., 2019; Guo et al., 2023). This finding may explain why many earlier studies could only explain a portion of the variation in phytoplankton communities using environmental factors alone.

The present study focuses on the distribution, composition, and assembly patterns of prokaryotic and eukaryotic phytoplankton, as well as their potential relationship with human activities in the waters from the PRE to cold seep in the near waters of Xisha Islands. We aimed to determine (1) the distribution, diversity, and abundance of phytoplankton in the study area, (2) the potential impact of cold seep on phytoplankton structure and composition, (3) the assembly mechanisms of the phytoplankton community, and (4) the effects of environmental parameters, location, and depth on the phytoplankton structure.

## 2 Materials and methods

### 2.1 Sampling

**Figure 1:**
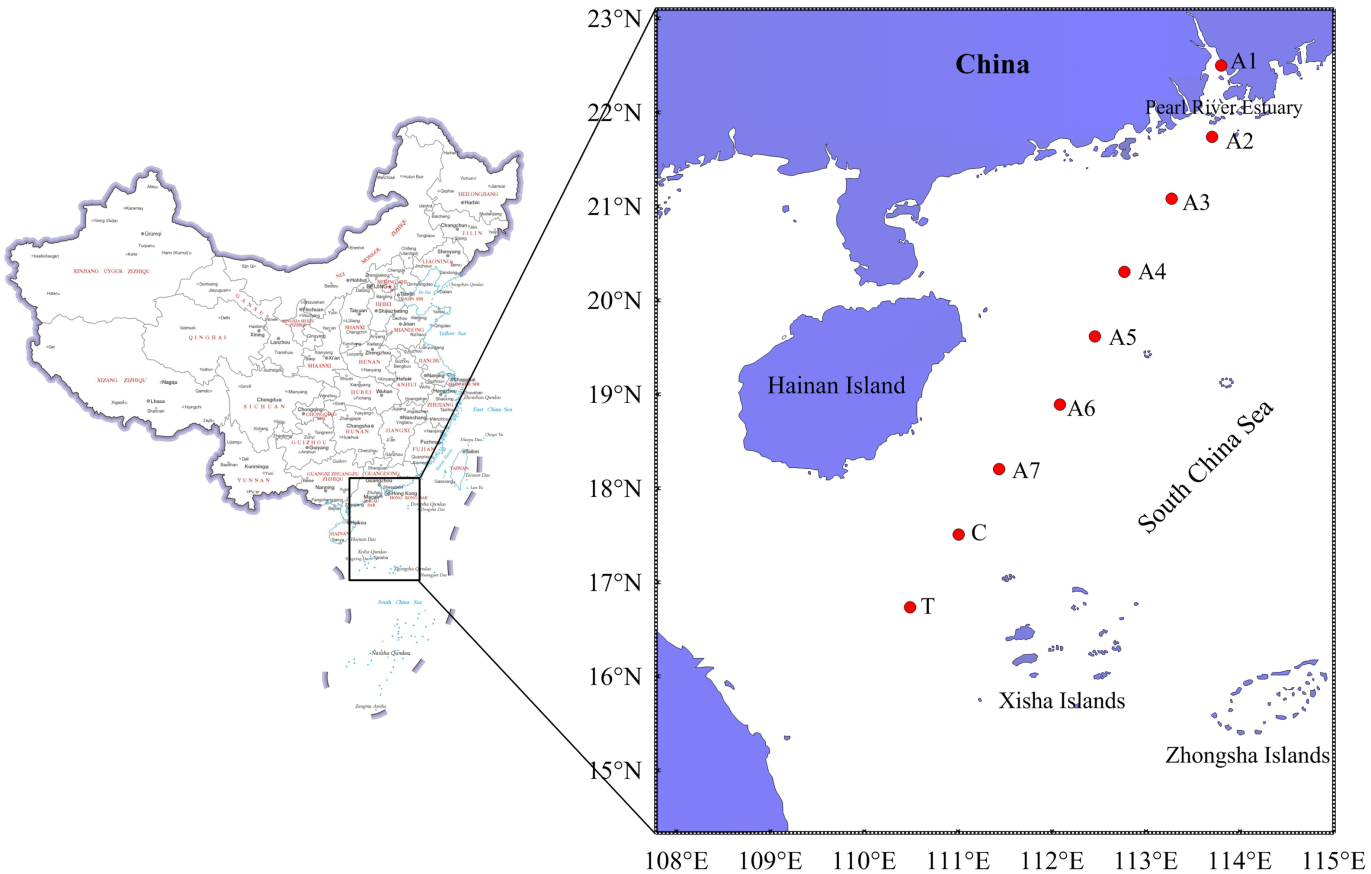
Map showing the investigated stations from the Pearl River Estuary to a cold seep in the northern South China Sea.

Nine stations were investigated for marine biodiversity in a cold seep during August 2023 by the “Nan Feng” ship. These stations were from PRE to the cold seep in the NSCS (Figure 1). Four areas, including PRE (Station A1, Station A2, and Station A3), the central area (CR, Station A4 to A7), the control area of the cold seep (Station C), and the cold seep (Station T), were divided for subsequent analysis. Eleven depths, including 0 m, 25 m, 50 m, 75 m, 150 m, 250 m, 500 m, 750 m, 1,200 m, and 1,400 m, were investigated in both Station T and C, and the euphotic layer (≤250 m) was studied in PRE and the central area. 4.5 L of seawater was collected at each depth using a conductivity-temperature-depth carousel multisampling system (CTD). Meanwhile, temperature and salinity were measured using the temperature sensor and salinity sensor, respectively. Dissolved oxygen and pH were measured using a YSI multi-parameter water quality sensor (YSI Plus, United States). The acid Lugol’s solution (final concentration, 1.5 ~ 2% v/v) was added to the 2 L surface water samples (0 m) of each station.

### 2.2 Sample processing and cell counting

One liter seawater samples were filtered for the measurement of chlorophyll a (Chl a) concentration using 0.7 μm GF/F membranes (Whatman, Maidstone, England). The concentrations of Chl a were confirmed by UV spectrophotometry based on the Specification for marine monitoring Part 4: Seawater analysis. Additionally, 1.5 L seawater samples for phytoplankton identification were filtered using 0.45 μm membranes (Millipore, Billerica, MA, United States). The filtered membranes were carefully transferred into 5 mL sterile tubes and stored immediately in liquid nitrogen. The filtered waters were stored at −20°C and subsequently determined the concentrations of nitrate (NO_3_), nitrite (NO_2_), ammonium (NH_4_), phosphate (PO_4_), silicate (SiO_3_), total nitrogen (TN), and total phosphorus (TP) (Bendschneider and Robinson, 1952; Mullin and Riley, 1955; Murphy and Riley, 1962; Sagi, 1966; Ebina et al., 1983).

Surface phytoplankton samples fixed with acid Lugou’s solution were transferred to the laboratory and concentrated based on the method of Utermohl (1958). Microalgae were counted using a microscope at 100× magnification.

### 2.3 DNA extraction, amplification, and sequencing

The filters containing phytoplankton were used to extract total DNA using the Power Soli^©^ DNA Extraction Kit (MOBIO, United States) according to the manufacturer’s instructions. The genomic DNA was examined using 1% agarose gel electrophoresis. The V4 region of 18 s rDNA was amplified using universal primers for eukaryotes: 3NDF forward primer 5′-GGCAAGTCTGGTGCCAG-3′ and V4-euk-R2R: 5′-ACGGTATCTRATCRTCTTCG-3′ (Mora et al., 2019). The procedures of polymerase chain reaction (PCR) were as follows: 3 min at 95°C for initialization, 35 cycles of 30 s denaturation at 95°C, 30s annealing at 55°C and 45 s extension at 72°C, and final extension at 72°C with 10 min. The cyanobacteria-specific primers CYA359F (5′-GGGGAATYTTCCGCAATGGG-3′)/CYA781R (5′-ACTACWGGGGTATCTAATCCC-3′) were used to amplify the V4 regions of 16 s rDNA in cyanobacteria (Monchamp et al., 2018). The PCR procedures were as follows: initial denaturation at 95°C for 10 min, followed by 35 cycles of denaturation at 95°C for 15 s, annealing at 60°C for 30 s, and extension at 72°C for 45 s, with a final extension at 72°C for 5 min. PCR was replicated three times for each sample, and the products were pooled into one sample. The pooled samples were purified using the AxyPrep DNA Purification Kit (AxyGEN, USA), eluted with 10 mmol L^−1^ Tris–HCl, and tested with 2% agarose gel electrophoresis. The PE300 sequencing of gene libraries was conducted using the TruSeq^™^ DNA Sample Prep Kit (Illumina, San Diego, CA, United States) following the manufacturer’s instructions. The libraries were sent to Majorbio Bio-Pharm Technology Co., Ltd. (Shanghai, China), and sequenced on a Miseq Illumina platform.

### 2.4 Bioinformatics processing

The HTS data were pre-processed using QIIME v. 1.9.1 (Edgar, 2010). Sequences with low-quality scores (below 25) and those shorter than 200 bp were discarded. The adaptor sequences were removed using USEARCH v. 10 (Edgar, 2013). For 18S rDNA, a total of 3,109,658 raw reads were obtained. Paired-end reads were successively split, filtered, and assembled. The DADA2 denoising method was employed to optimize the data and a total of 1,810,818 clean sequences were generated. The sequences with 100% similarity were clustered into the same amplicon sequence variants (ASVs) using the Uclust algorithm and annotated based on the SILVA 138 database. Among them, ASVs of zooplankton (9,711) and fungi (318) in 18 s rDNA were filtered out, and ASVs of eukaryotic phytoplankton were rarefied to a unified read count using the DESeq method for subsequent analysis. In addition, a total of 1,913,076 raw reads of 16S rDNA were obtained. After applying the DADA2 denoising method, 1,267,154 clean sequences were generated. Sequences with 100% similarity were clustered into ASVs and annotated using the NT database. The cyanobacteria-specific ASVs were selected and rarefied for downstream analysis. The raw data have been submitted to the National Center for Biotechnology Information (NCBI) with the accession numbers PRJNA1125195 and PRJNA1125182 for eukaryotic and prokaryotic phytoplankton, respectively.

### 2.5 Data analysis

The dominant species of phytoplankton were determined by dominance (*Y* > 0.02) based on the results of cell counting (Xu and Chen, 1989). The alpha diversity index of eukaryotic and prokaryotic microalgae including Shannon diversity index (*H′*), Pielou’s evenness index (*J’*), and Margalef richness index (*D*) were determined using Primer v. 7. Kruskal-Wallis non-parameter test was conducted to determine the significant differences of diversity index between different areas using SPSS Statistic 25.0. Principal coordinate analysis (PCoA) combined with permutation multivariate analysis of variance (PERMANOVA) based on Bray–Curtis dissimilarity was conducted to confirm the beta diversity of phytoplankton between different areas and between diverse depths. The relationships between environmental factors and phytoplankton in the photic zone were analyzed using redundancy analysis (RDA). In addition, the correlations between the alpha diversity index and environmental factors were examined using the Spearman correlation test. NCM was employed to predict the occurrence frequency of ASVs and determine the relative importance of stochastic processes in the community assembly of phytoplankton. Furthermore, a null model was operated to distinguish assembly processes, including homogeneous selection, heterogeneous selection, dispersal limitation, homogeneous dispersal, and ecological drift (undominated process) (Stegen et al., 2013). The beta nearest taxon index (βNTI) and the Raup-Crick index based on Bray-Curtis (RC_bray_) were calculated to quantity the assembly processes. The value of βNTI less than −2 represented homogeneous selection, while the values greater than 2 indicated heterogeneous selection. For βNTI values between −2 and 2, RC_bray_ values > 0.95 and < −0.95 represented dispersal limitation and homogenizing dispersal, respectively. RC_bray_ values between −0.95 and 0.95 indicated undominated processes (Zhou and Ning, 2017). All analysis and visualization were performed using the online tools provided by the Majorbio Cloud Platform, unless otherwise specified.

## 3 Results

### 3.1 Environmental factors

Thirteen environmental factors were measured to explore the key factors driving the distribution of phytoplankton in the NSCS (Supplementary Figure S1). The following descriptions refer to environmental data above a depth of 150 m unless otherwise specified. Temperature ranged from 15.37 to 30.92°C, with an average value of 25.01°C. The mean temperature below 150 m was 3.18°C (Supplementary Figure S1A). Salinity, DO, and pH ranged from 12.18 (Station A1) to 34.56 (Station A6 at 150 m), from 3.68 (Station A1) to 7.44 mg L^−1^ (Station A2 in surface water), and from 7.91 (Station A6 in surface water) to 8.45 (Station T at 75 m), respectively (Supplementary Figures S1B–D). The concentrations of nutrients varied significantly in the vertical distributions in the study area. The concentrations of nitrate, nitrite, ammonium, TN, silicate, phosphate, and TP were between 0.13 and 37.48 μmol L_−1_, between 1.51 and 11.85 μmol L^−1^, between 0.07 and 28.34 μmol L^−1^, between 11.99 and 67.33 μmol L^−1^, between 0.15 and 77.32 μmol L^−1^, between 0.11 and 1.68 μmol L^−1^, and between 0.01 and 1.51 μmol L^−1^, respectively (Supplementary Figures S1F–K). The maximum value of Chl a was observed at Station A1, with a value of 0.08 μg L^−1^, and the average concentration was 0.01 μg L^−1^ (Supplementary Figure S1L). The concentrations of nitrate, ammonium, and silicate were statistically different between different stations (ANOVA, *p* < 0.05). There were higher concentrations of nitrite and TP in the cold seep station (Station T) than in the control station (Station A8, Mann–Whitney U test, *p* < 0.05).

### 3.2 Phytoplankton community

#### 3.2.1 Community composition and structure

**Figure 2:**
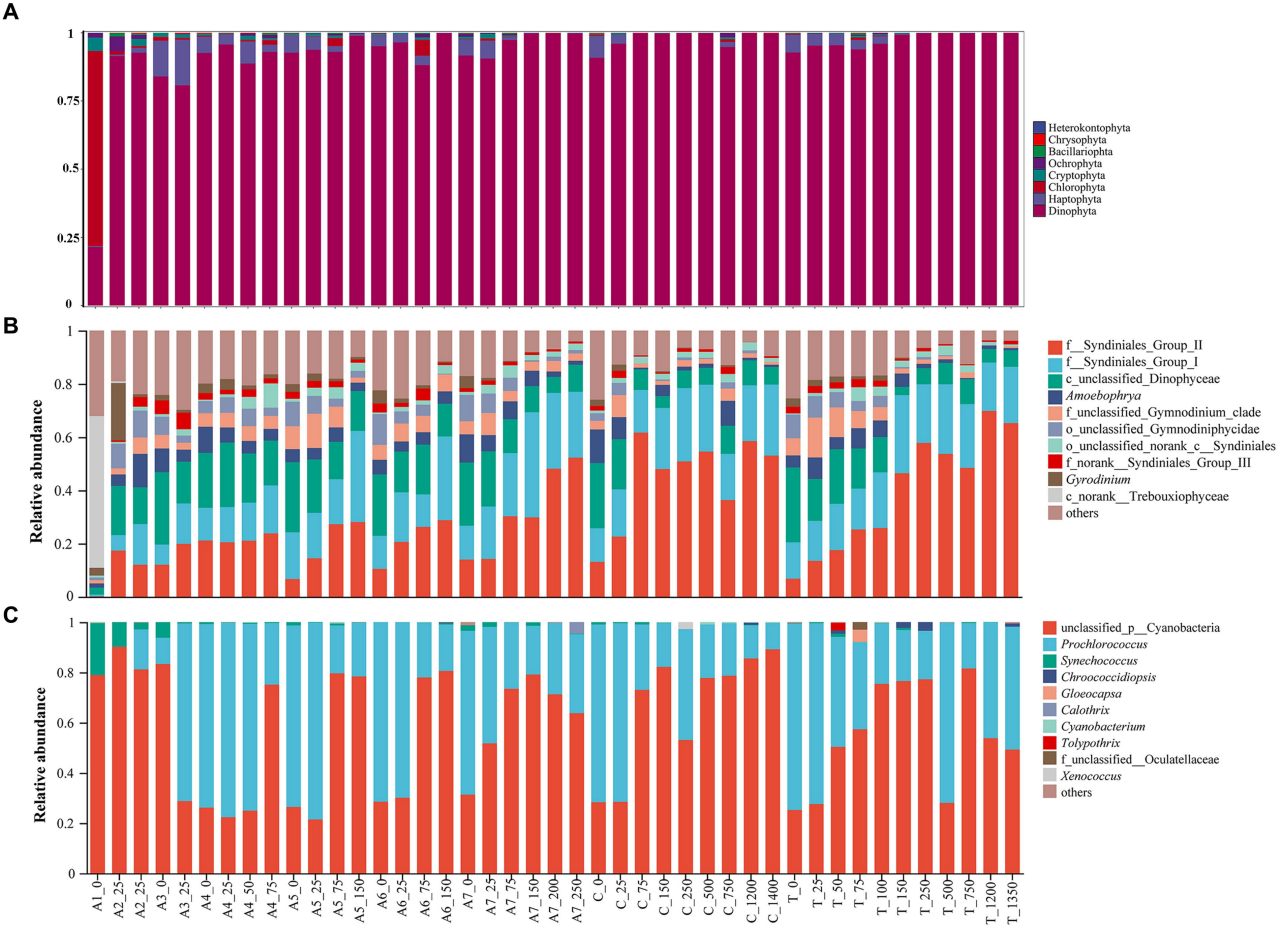
Histogram showing relative abundance of eukaryotic phytoplankton on phylum level **(A)**, on eukaryotic genus level **(B)** and on cyanobacteria genus level **(C)**.

Seven hundred and twenty-two ASVs belonging to Cyanophyta were detected based on 16S rDNA HTS analysis, and 7,181 ASVs for eukaryotic microalgae were detected in the NSCS according to 18S rDNA sequencing. The eukaryotic ASVs could be categorized into eight phyla: Dinophyta, Haptophyta, Chlorophyta, Cryptophyta, Ochrophyta, Bacillariophyta, Chrysophyta, and Heterokontophyta (Figure 2A). Among them, Dinophyta had 11,770 ASVs and was the most dominant phylum in all stations of the NSCS, accounting for 92.17% of the eukaryotic ASVs. Haptophyta and Chlorophyta accounted for 3.34 and 1.81%, respectively. The remaining five phyla accounted for below 1% of the eukaryotic ASVs, ranging from 8 to 67 ASVs (Figure 2A). Dinophyta predominated in all stations of the NSCS except in Station A1, which was dominated by Chlorophyta, accounting for 47.80%.

The top 10 abundant genera of eukaryotic phytoplankton belonged to Dinophyta, except for Trebouxiophyceae (class level) at station A1, accounting for 52.95% of the total relative abundance. Syndiniales (order level) predominated in eukaryotic microalgae and showed dominant relative abundance (Figure 2B). In addition to unclassified cyanobacteria, *Prochlorococcus* showed the highest relative abundance in cyanobacteria of all samples (Figure 2C), ranging from 0.00 to 78.42%.

**Figure 3:**
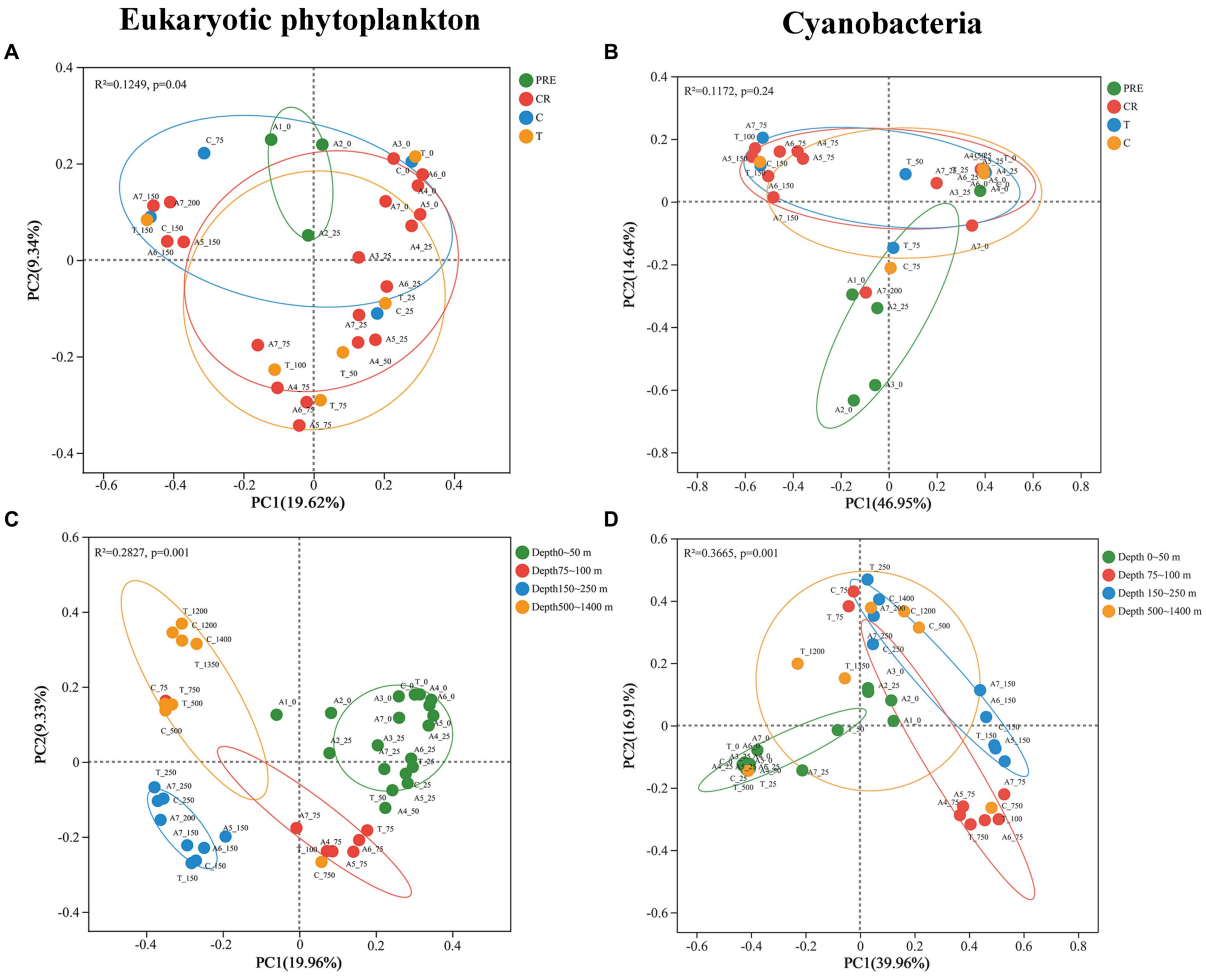
The results of principal coordinate analysis (PCoA) for eukaryotic phytoplankton (left) and cyanobacteria (right) across four areas **(A,B)** and different depths **(C,D)**.

PCoA based on Bray-Curtis distance was conducted to assess the horizontal and vertical variations of the phytoplankton community in the NSCS. The results revealed significant differences in the horizontal distributions of eukaryotic phytoplankton (PERMANOVA, *p* < 0.05; Figure 3A), whereas no significant differences in cyanobacteria were found (PERMANOVA, *p* > 0.05; Figure 3B). When observed vertically, obvious differences were found in the distribution of eukaryotic phytoplankton across four depth zones. Different microalgae groups can be found at different depths (PERMANOVA, *p* < 0.05; Figure 3C). In addition, significant differences were observed in the distribution of cyanobacteria between the surface waters (0 ~ 50 m depth), waters at 75 ~ 100 m depth, and waters at 150 ~ 250 m depth, excluding the deepest waters (depths 500 ~ 1,400 m; Figure 3D).

#### 3.2.2 Alpha diversity

**Figure 4:**
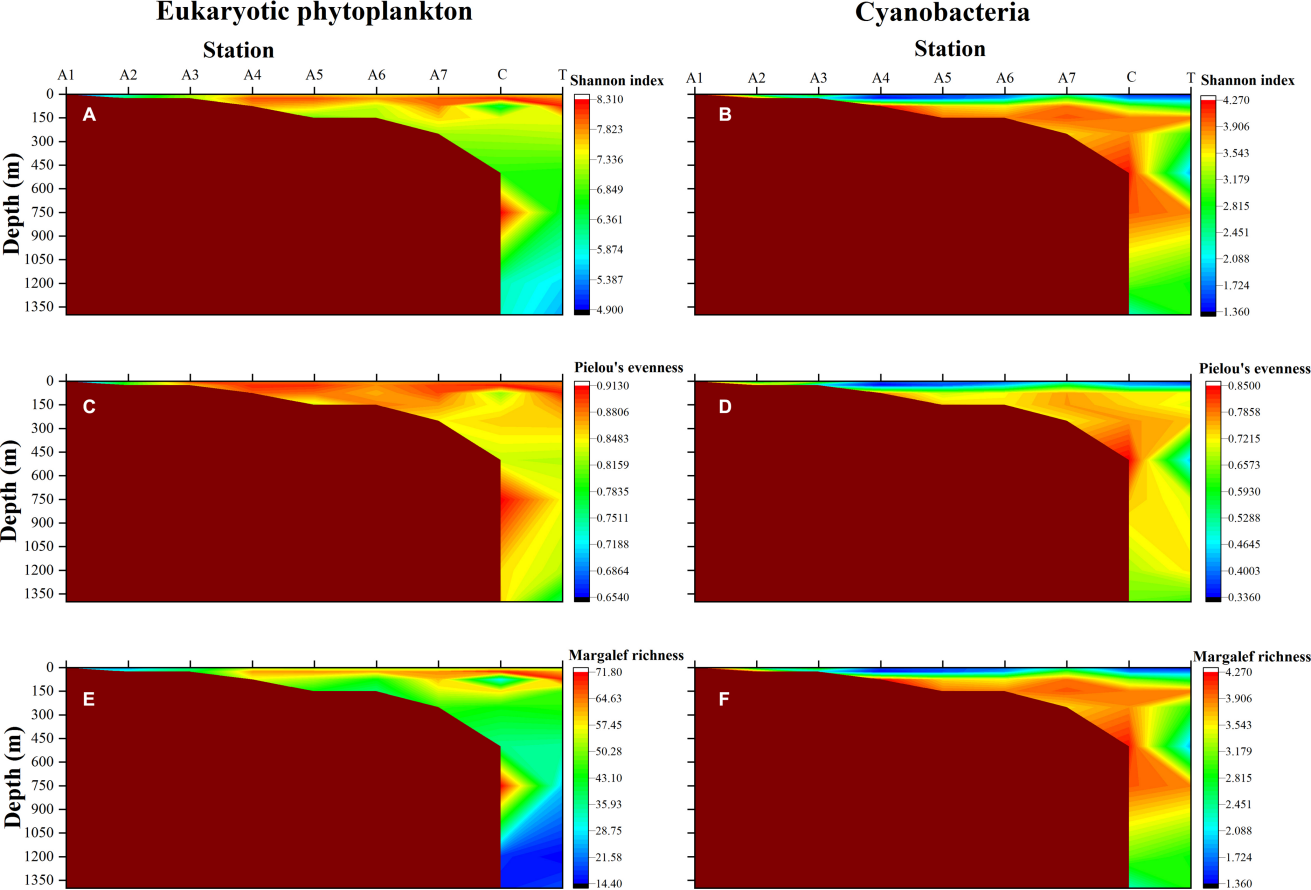
The vertical distributions of alpha diversity index of eukaryotic phytoplankton (left) and cyanobacteria (right). **(A,B)** Shannon-Wiener diversity index; **(C,D)** Pielou’s evenness index; **(E,F)** Margalef richness index.

The data observed by HTS was used to reveal the distribution of alpha diversity in the waters of the NSCS. Horizontally, the mean values of Shannon diversity, Pielou’s evenness, and Margalef richness of eukaryotic microalgae in the photic zone were 7.40, 0.87, and 50.29, respectively (Figures 4A,C,E). The three alpha indices of eukaryotic phytoplankton in the PRE station (A1 and A2) were significantly lower than other stations (Kruskal-Wallis test, *p* < 0.05). The Shannon index ranged from 4.91 (Station A1) to 8.19 (Station T at 75 m) in the photic zone. Vertically, both Shannon diversity and Margalef richness index decreased with increasing depth in general, with maximum values occurring at 25 m (Figures 4A,F). However, Pielou’s index showed a similar distribution pattern at different depths (Figure 4C). The distribution patterns of the alpha diversity index in cyanobacteria were different from the eukaryotic algae. The PRE Station A1 showed relatively high values in Shannon index (4.17), Pielou’s index (0.72), and Margalef index (7.28), and all three indices exhibited high values between 75 m and 750 m (Figures 3B,D,F).

### 3.3 Composition and abundance of phytoplankton

**Figure 5:**
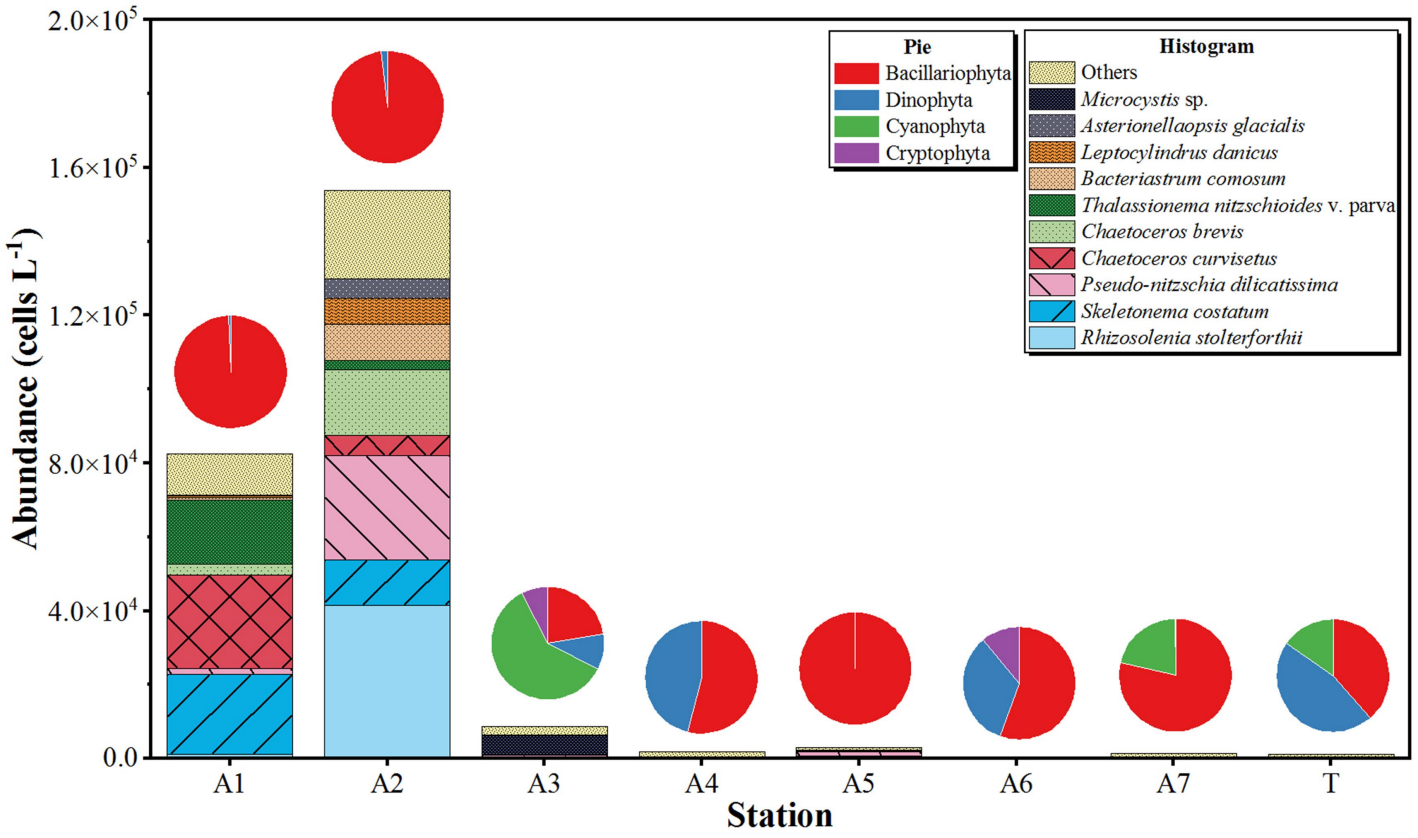
The spatial distribution of phytoplankton abundances based on the results of microscopic observation in northern South China Sea during August 2023. Histogram showing the abundance of top 10 species of phytoplankton and the pie showing the relative abundance of phytoplankton phylum.

The results of phytoplankton composition and abundance based on optical microscopic observation differed from the results of HTS. In August 2023, a total of 47 species of phytoplankton were found in the NSCS surface water. They were composed of Bacillariophyta (37 species, 80.85%), Dinophyta (six species, 12.77%), Cyanophyta (two species, 4.26%), and Cryptophyta (one species, 2.13%). Ten dominant species of phytoplankton were identified, including *Asterionellaopsis glacialis*, *Bacteriastrum comosum*, *Chaetoceros brevis*, *Chaetoceros curvisetus*, *Leptocylindrus danicus*, *Pseudo-nitzschia dilicatissima*, *Rhizosolenia stolterforthii*, *Skeletonema costatum*, *Thalassionema nitzschioides* v. parva, and *Microcystis* sp. Among them, *R. stolterforthii*, *S. a costatum,* and *P. dilicatissima* were the most abundant species, with abundances of 5.43 × 10^3^, 4.38 × 10^3^, and 3.90 × 10^3^ cells L^−1^, respectively (Figure 5).

The abundance of phytoplankton in surface water was counted based on microscopic observations, with an average value of 3.16 × 10^3^ cells L^−1^. The abundances of microalgae ranged between 5.83 × 10^2^ and 1.54 × 10^5^ cells L^−1^, with the minimal and maximal abundances recorded at station A6 and station A2, respectively (Figure 5). Bacillariophyta was the most abundant phylum, accounting for 95.18% of the total abundance with an average abundance of 3.01 × 10^4^ cells L^−1^. The minimal and maximal abundances similarly occurred at stations A6 (3.24 × 10^5^ cells L^−1^) and A2 (1.51 × 10^5^ cells L^−1^). The second most abundant taxa was Dinophyta, contributing to 0.00 to 46.15% of phytoplankton abundance in each station. No Dinophyta were found in stations A5 and A7, with the highest abundance observed at station A2, reaching 2.86 × 10^3^ cells L^−1^ (Figure 5). Cyanophyta and Cryptophyta were sporadically distributed in the NSCS with abundances below 1.0 × 10^3^ cells L^−1^, except for the abundance of Cyanophyta in station A3 (5.20 × 10^3^ cells L^−1^, Figure 5).

### 3.4 The relationships between environmental factors and phytoplankton community

**Figure 6:**
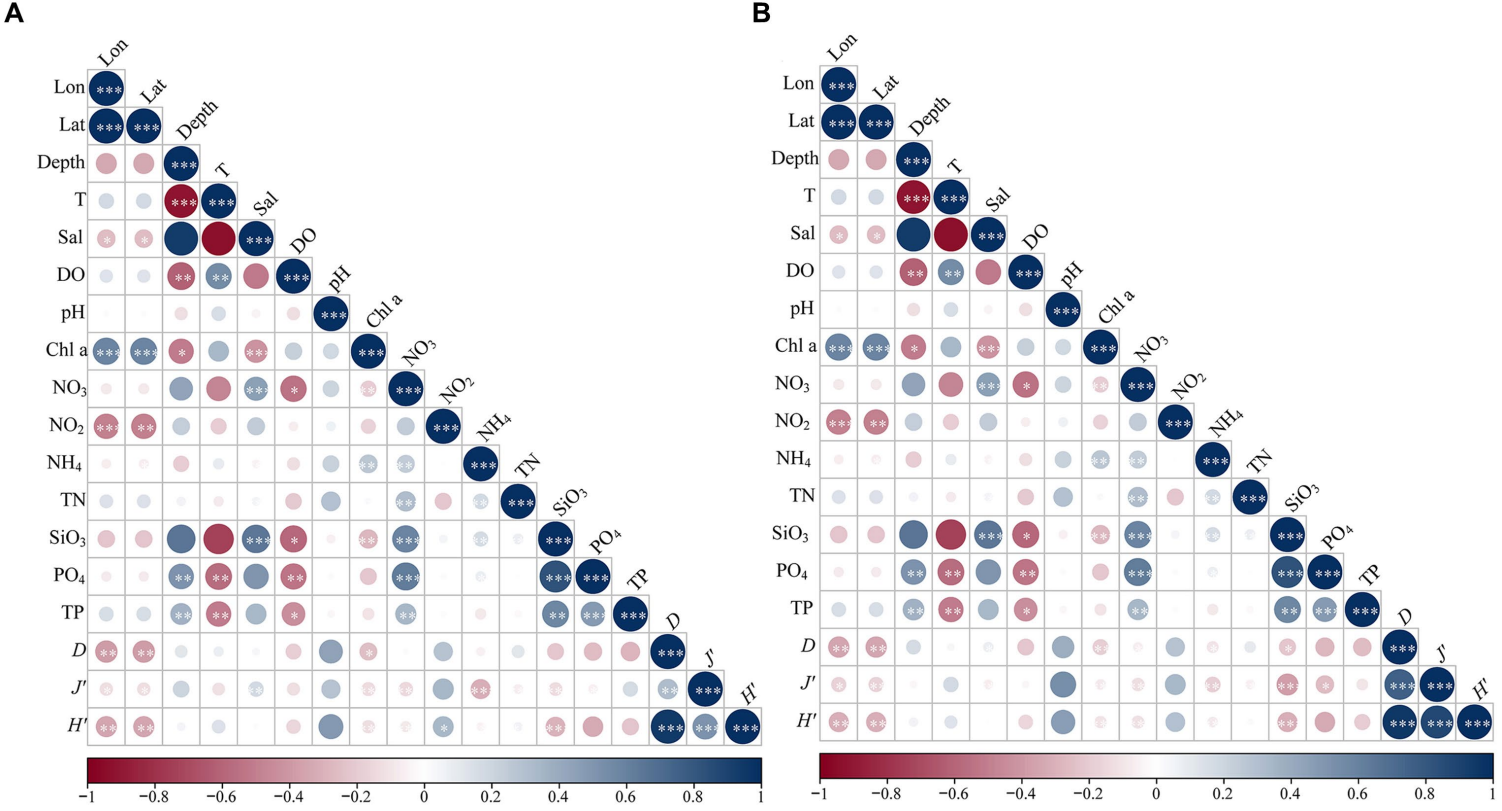
Correlation heatmap showing the relationships between Shannon-Wiener diversity index (*H′*), Pielou’s evenness index (*J’*), Margalef richness index (*D*) of eukaryotic **(A)** and prokaryotic **(B)** phytoplankton and environmental parameters in northern South China Sea during August 2023. Blue and red cycles indicate positive and negative correlations, respectively. The existences of cycle represent *p* < 0.05, and the sizes of cycle indicate the value of Spearman’s coefficient.

A Spearman’s correlation was conducted to explore the relationships between the Shannon-Wiener diversity index (*H′*), Pielou’s evenness index (*J’*), Margalef richness index (*D*) of phytoplankton and environmental parameters. The results revealed that the Shannon-Wiener index and Margalef index of eukaryotic microalgae were positively correlated with the concentrations of nitrite and salinity, respectively (Figure 6A; Supplementary Table S1). On the contrary, the three alpha-diversity indices were significantly negatively related to longitude, latitude, and concentrations of Chl a. Furthermore, the indices of diversity and evenness decreased with increasing concentrations of nitrate, ammonium, and silicate (Figure 6A; Supplementary Table S1). The alpha diversities of cyanobacteria were significantly positively correlated with depth, concentrations of nitrate, silicate, phosphate, and TP (Supplementary Table S2). The diversity indices were clearly negatively related to temperature and dissolved oxygen, except for the lack of correlation between Pielou’s evenness index and the concentration of nitrate (Figure 6B). Additionally, the Shannon-Wiener diversity index increased as the salinity increased (Figure 6B).

**Figure 7:**
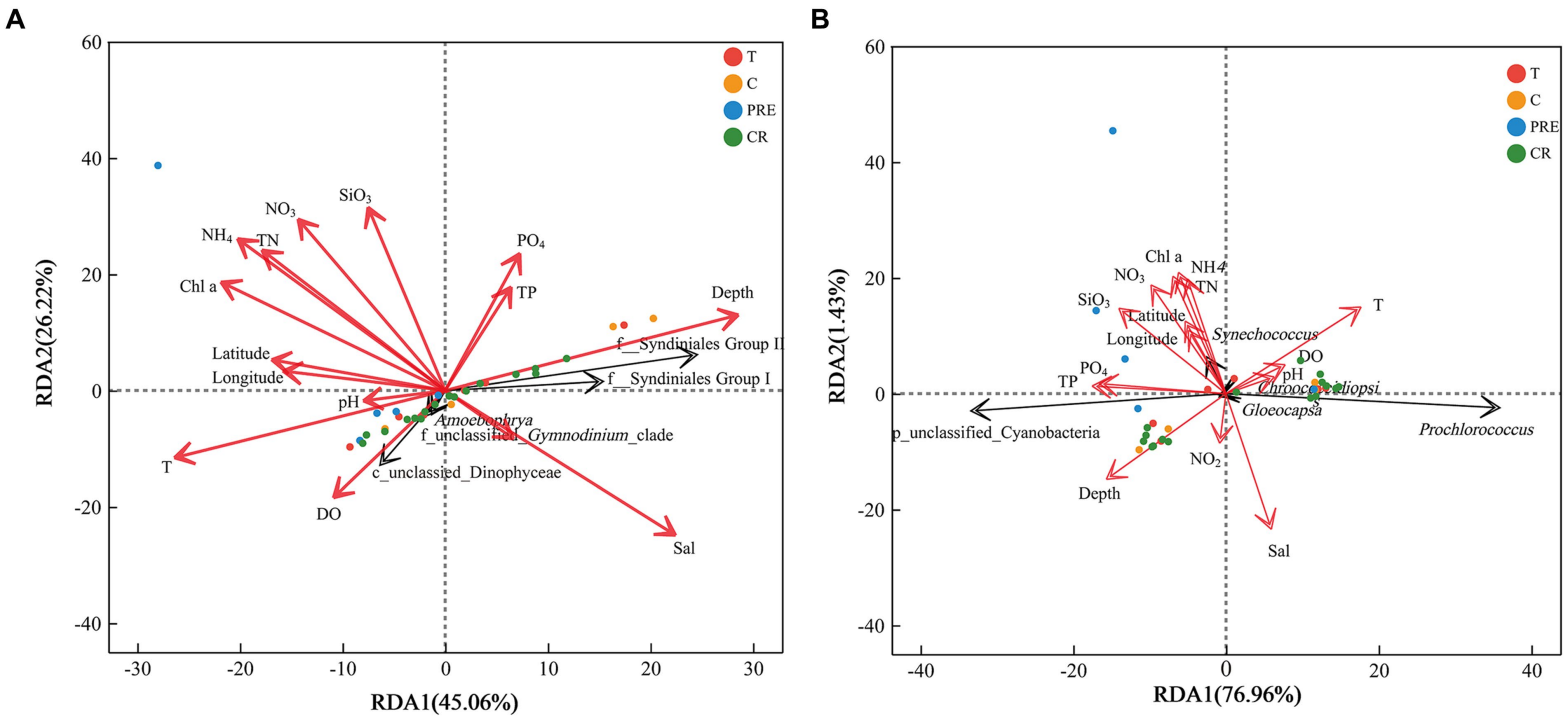
Redundancy analysis showing the relationships between environmental factors and phytoplankton genera in the northern South China Sea during August 2023. **(A,B)** were the results of eukaryotic and prokaryotic phytoplankton, respectively. Red and black arrows represent environmental factors and top five genera, respectively, and cycles represent the samples.

Subsequently, RDA was performed to explore the influences of environmental factors on the phytoplankton community. The first two axes of eukaryotic phytoplankton and cyanobacteria explained 71.26 and 78.39% of the total variance, respectively (Figure 7). Overall, the distributions of eukaryotic microalgae in the NSCS were significantly influenced by environmental factors. The relative abundance was significantly correlated with the factors, excluding longitude, pH, and the concentration of nitrite (Supplementary Table S3). Among them, the top two genera (f_Syndiniales_Group I and f_Syndiniales_Group II) of eukaryotic phytoplankton were positively influenced by depth and salinity, and negatively related to temperature, DO, and nutrient concentrations (Supplementary Table S4). However, the other genera (c_unclassified and *Amoebophrya*) showed opposite correlations. In addition, the prokaryotic microalgae cyanobacteria (unclassified_p_Cyanobacteria) were positively impacted by depth, silicate, phosphate, and total phosphorus, while the relative abundance of *Prochlorococcus* was positively correlated with temperature (Figure 7B).

### 3.5 Assembly of phytoplankton community

**Figure 8:**
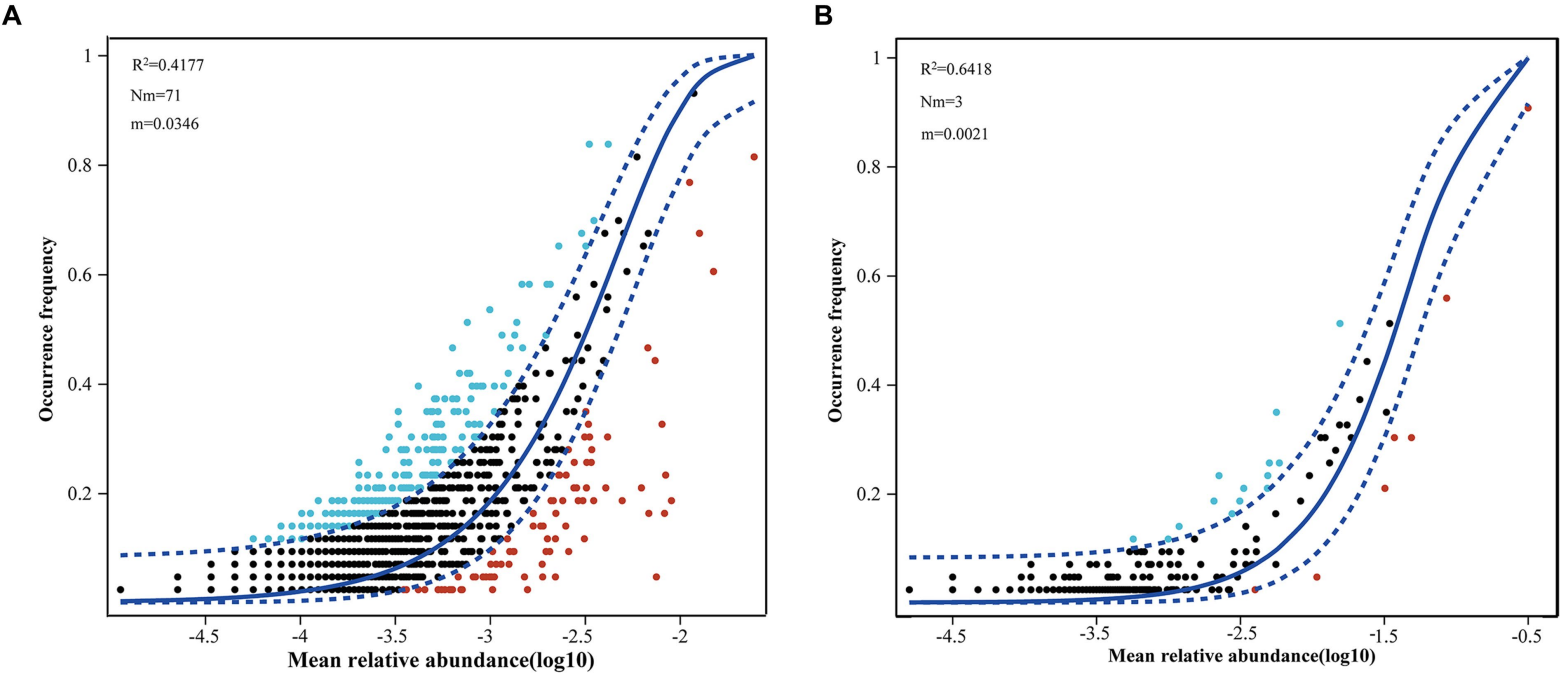
Fit of neutral community model to eukaryotic **(A)** and prokaryotic **(B)** phytoplankton ASVs data in the northern South China Sea during August 2023.

**Figure 9:**
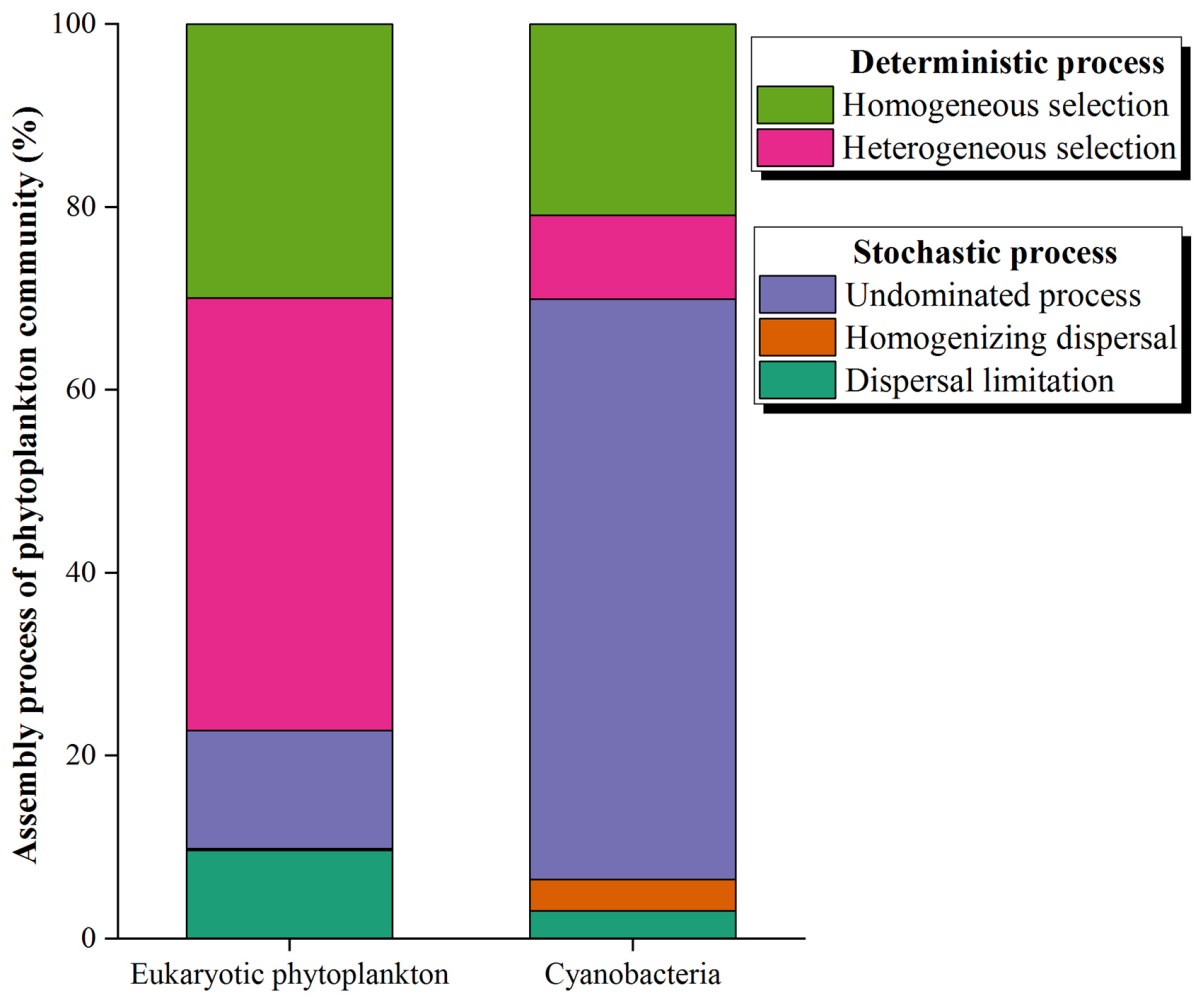
Null model showing the contributions of different ecological processes to the assembly of phytoplankton in the northern South China Sea during August 2023.

More than 20% of the variances were left unexplained based on the RDA between phytoplankton and environmental factors. A NCM was conducted to explore the role of stochastic processes in the distributions of phytoplankton. The NCM better fit the phytoplankton community, with R squared values of 41.77 and 64.18% for eukaryotic microalgae and cyanobacteria, respectively (Figure 8), indicating that stochastic processes play a nonnegligible role in the assembly of the phytoplankton community in the NSCS. Furthermore, a null model further explored the relative importance of random processes and deterministic processes. The results revealed that deterministic processes played a more important role in community assembly, with percentages of 77.22% for eukaryotic phytoplankton (Figure 9). Meanwhile, stochastic processes also made a non-negligible contribution to the assembly of the community. Among the stochastic processes, dispersal limitation (9.63%) and undominated processes (ecological drift, 12.93%) were the main processes affecting eukaryotic phytoplankton. However, the stochastic process (69.93%) dominated community assembly of cyanobacteria and the undominated process accounted for 63.44% of the total assembly process (Figure 9).

## 4 Discussions

### 4.1 The cold seep supports the growth and diversity of cyanobacteria

The influence of cold springs on chemolithoautotrophic organisms such as bacteria and chemosynthetic bivalves attracted attention (Levin, 2005), but few studies explored the impact of deep-sea cold seeps on phytoplankton. Some species of microalgae have a heterotrophic lifestyle (Zhang et al., 2021). The methane in cold seeps provides carbon for chemosynthetic phytoplankton, especially cyanobacteria, which may, in turn, serve as a nitrogen source for attached bivalves (Li et al., 2020). In the present study, the impact of cold seeps on the composition and diversity of prokaryotic and eukaryotic phytoplankton was explored based on HTS. The results showed that no significant differences were found in ASV numbers, alpha and beta diversities between the cold seep (site T) and the control area (site C). However, Venn analysis revealed that there were more specific genera of phytoplankton (32) at the cold seep site compared to the control site (12; Supplementary Figure S2), suggesting that the cold seep site has higher biodiversity (Diao et al., 2023). The Shannon-Wiener diversity of eukaryotic phytoplankton gradually decreased from surface to bottom in cold seep site, which is similar to the findings described by Diao et al. (2023). The top 10 genera of eukaryotic phytoplankton belonged to Syndiniales and accounted for 98.32% in relative abundance at 1,200 m depth of site T, which may be explained by the parasitic lifestyle of Syndiniales (Stoeck et al., 2006). Surface phytoplankton was predominantly composed of Syndiniales, and combining previous studies, it is inferred that deep-sea eukaryotic Syndiniales at cold seeps originate from sinking cells (Stoeck et al., 2006). Interestingly, we found that the Shannon, Pielou’s, and Margalef indices of prokaryotic microalgae increased with depth, suggesting that the fluids of cold seeps may support a more diverse prokaryotic community. As indicated by the Venn analysis, there were significantly more specific genera of prokaryotic phytoplankton at Site T compared to the control area, indicating an increase of genera with depth. Furthermore, in addition to the unclassified cyanobacteria, *Prochlorococcus* accounted for more than 50% of the relative abundance in prokaryotic phytoplankton in cold seeps. Some previous studies have found that certain species of *Prochlorococcus* exhibit a heterotrophic lifestyle to obtain sufficient carbon and provide nitrogen to attached bivalves in deep-sea cold springs (Pile and Young, 1999; Stoeck et al., 2006). In conclusion, eukaryotic algae in the outflows of cold seep may originate from sinking cells in the surface waters (Stoeck et al., 2006; Diao et al., 2023), while the growth and diversity of prokaryotic organisms are supported by the cold seep fluids. Further field and lab studies are necessary to investigate the effects of cold seep fluids on eukaryotic and prokaryotic phytoplankton.

### 4.2 The distribution, abundance, and structure of phytoplankton in the waters from PRE to Xisha Islands

Phytoplankton are the main photosynthetic organisms living in the euphotic zone, contributing approximately 50% of carbon in the world. In this study, the species numbers and abundances of phytoplankton gradually decreased from PRE to the adjacent waters of Xisha Islands based on HTS and morphological observation. The maximal concentrations of phytoplanktonic pigment were observed in coastal waters in the summer in the NSCS (Ho et al., 2015). The distribution patterns of phytoplankton abundance in the NSCS can be easily explained. From PRE to the Xisha Islands, the influence of human activities gradually decreases, and nutrient concentrations also gradually decrease, resulting in differences in the distribution of phytoplankton abundance that are predominantly influenced by nutrients. We found that Bacillariophyta dominated the phytoplankton community, accounting for 80.85%, followed by Dinophyta at 12.77%, based on morphological observation. Peng et al. (2023) identified a total of 29 species in the shallow waters of the Xisha Islands, with diatoms accounting for 79% with 23 species, and dinoflagellates as the second-largest group at 17% (Peng et al., 2023). Additionally, the maximum abundance (1.54 × 10^5^ cells L^−1^) was observed at station A2, higher than at station A1 (8.24 × 10^4^ cells L^−1^) located inside the PRE, possibly due to the more suitable salinity conditions at station A2 (30.98–34.28) compared to station A1.

Previous studies often found that diatoms are the dominant group in the phytoplankton community based on morphological observation in the NSCS (Wei et al., 2018), which is consistent with the morphological results of this study. However, based on HTS technology, the present study found that Dinophyta had the highest number of ASVs, accounting for 92.17%, while diatoms accounted for only 0.63%. Peng et al. (2023) studied the composition of phytoplankton communities during the outbreak of Crown-of-Thorns Starfish in the Xisha Islands area. The results showed 1,176 operational taxonomic units (OTUs) of eukaryotic phytoplankton, primarily dominated by dinoflagellate with 812 OTUs, accounting for 69%, and diatoms only accounted for 14% (Peng et al., 2023). A cruise using HTS investigated the composition of phytoplankton in the waters near Hainan Island during August 2016. The results showed that phytoplankton were mainly composed of Dinophyta and Syndiniales (including Group I and II), accounting for 17.09 and 21.12%, respectively (Gong et al., 2020). We found the highest relative abundance of genera belonged to parasitic Syndiniales which are widespread in all marine waters (Clarke et al., 2019). De Vargas et al. (2015) found parasitic Syndiniales dominated phytoplankton in an investigation of diversity and abundance based on HTS. In addition, the second largest group of cyanobacteria, *Prochlorococcus*, is a dominant photosynthetic organism in marine environments and contributes significantly to global photosynthesis (Biller et al., 2015). Previous research has found that *Prochlorococcus* is widely present in the coastal waters of the South China Sea (Jiao and Yang, 2002).

### 4.3 The assembly patterns of phytoplankton community

The NCM is based on neutral processes to infer the aggregation of stochastic processes in communities, and it is widely used in bacterial communities (Dini-Andreote et al., 2015) but less used in phytoplankton communities. The present study first explored the assembly patterns of the phytoplankton community in the South China Sea, China. Our results showed that stochastic processes accounted for 41.77 and 64.18% of the community assembly of eukaryotic and prokaryotic microalgae, respectively, suggesting that cyanobacteria were larger influenced by stochastic processes. Previous studies have reported that stochastic processes dominated in assembly of bacteria communities, including cyanobacteria, which are photosynthetic autotrophic microalgae (Zhang S. et al., 2022). Guo et al. (2023) found that the assembly of the bacterial community was driven by an undominated process, which is consistent with our results in the NSCS. The undominated process dominated cyanobacteria community, which may influenced by the complex flow patterns and anthropogenic activities (Rosindell et al., 2011). On the other hand, our results found that random processes accounted for 41.77% of the assembly of eukaryotic processes in the NSCS, which was inconsistent with the previous studies. Klais et al. (2017) determined that random processes dominated the assembly of phytoplankton communities in the Baltic Sea and only 24% of processes could not be explained by NCM. In addition, stochastic processes dominated the microeukaryotic community assembly in a subtropical river in China (Chen et al., 2019). The NCM explained 89.9, 88.5, and 89.6% of the community variation in the wet season, dry sea, and both seasons, respectively (Chen et al., 2019). The reason for the inconsistency is that environmental factors play an important role in community assembly. The RDA results showed that environmental factors could explain 71.26% of the variation in the eukaryotic microalgae community (Figure 7). The null model further demonstrated the significant role of the deterministic process (47.27% heterogeneous selection and 29.95% homogeneous selection) in the aggregation of eukaryotic microalgae communities. Recently, a study investigated the assembly processes of three main phytoplankton taxa (diatoms, *Synechococcus*, and Haptophyta) based on HTS data. The results also showed that heterogeneous selection dominated and accounted for over 60% (Xu et al., 2022). There was a lower relative abundance of environmental selection (50%) in the eukaryotic phytoplankton community assembly in the PRE compared to other areas (Supplementary Figure S3A), which may explain the significant differences in alpha and beta diversity of phytoplankton in the PRE (Xu et al., 2022).

### 4.4 Relationships between environmental factors and phytoplankton community

The Spearman’s correlation and RDA were conducted to investigate the impact of environmental factors on the distribution and diversity of phytoplankton communities in the NSCS. The results demonstrated a positive correlation between the Shannon index of eukaryotic microalgae and nitrite concentrations. The evenness index was positively correlated with salinity. However, all three indices were negatively correlated with latitude, longitude, ammonium, and silicate. Dinoflagellates were the dominant group in the phytoplankton community. Nitrite is a nutrient that dinoflagellate can directly absorb and utilize compared to the reduced states of nitrogen (NH_4_), and dissolved organic nitrogen (DON) is an important component of TN, accounting for an average of 47%. This may be the direct reason for the high biodiversity of dinoflagellates. Zhang W. et al. (2022) suggested that the high concentrations of DON enhanced the diversity of phytoplankton in the Qinhuangdao waters, Bohai Sea. Dinoflagellates have been proven to exhibit a mixotrophic mode of nutrition, allowing them to effectively utilize organic nutrients in the aquatic environment (Burkholder et al., 2008; Gobler, 2020). The RDA results indicated that environmental factors in the study area had a significant impact on the distribution of eukaryotic microalgae (*p* < 0.05), especially salinity, ammonium, depth, nitrate, silicate, TN, and temperature, with R-squared values all exceeding 0.6 (Figure 7A; Supplementary Table S3). Anderson and Harvey (2020) found a positive correlation between Syndiniales and temperature in the Skidaway River estuary in Georgia (Anderson and Harvey, 2020), and global warming has led to elevated water temperatures, which has constrained the ecological niche of non-flagellates in high-temperature environments (Hallegraeff, 2010; Xiao et al., 2018). Syndiniales are considered parasitic dinoflagellates (Anderson and Harvey, 2020), and a positive correlation between Syndiniales and *Gymnodinium* indicated a potential parasitic relationship between the two in the present study. Except in station A1 with a salinity of 12.18, the salinity at other stations is relatively high, with an average of 33.91, suitable for the growth of phytoplankton communities dominated by dinoflagellates. The relationships between nutrients and the distribution of microalgae communities are complex, and some previous studies investigating the relationship between phytoplankton and nutrient concentration have yielded different conclusions. Diao et al. (2023) found a positive relationship between dinoflagellate and DIP, but a study showed that high concentrations of DIP reduced the diversity and abundance of these microalgae (Zhang W. et al., 2022). In the present study, the study area showed high concentrations of nutrients and the average value of TN/TP was 70, suggesting phosphorus limitation. The RDA results found obvious correlations between phosphorus and the relative abundance of phytoplankton, suggesting the concentrations of phosphorus may be a key factor influencing the distribution of eukaryotic phytoplankton in the NSCS.

Our results showed cyanobacteria widely distributed across various sampling sites, especially in bottom samples. Cyanobacteria dominated deep water bodies (Figure 2A) due to their unique characteristics, including colony formation, toxin production, and nitrogen fixation (Wang et al., 2021). The RDA results showed unclassified cyanobacteria were positively correlated with silicate, phosphate, TP, and depth. Temperature, salinity, water stability, and the availability of nitrogen and phosphorus are the main factors controlling the cyanobacterial community in the Baltic Sea (Wasmund et al., 2012; Andersson et al., 2015). Among them, temperature and salinity were the main factors driving the distribution of cyanobacteria community in summer (Bertos-Fortis et al., 2016). Our results indicated that a portion of cyanobacterial distribution was unexplained by environmental factors in the NSCS, as stochastic processes played a significant role, accounting for 64.18% of the community assembly process. *Prochlococcus* was the main taxa of cyanobacteria and was positively related with temperature but negatively with phosphorus and silicate. Temperature was the main factor influencing the abundance and distribution of *Prochlorococcus* (Wu et al., 2018). Jiao and Yang (2002) found that the activity of major ocean currents in Chinese coastal waters, such as the Kuroshio and Taiwan Warm Current, as well as changes in water temperature, were the main causes of seasonal differences in *Prochlorococcus* distribution. The distribution of *Prochlorococcus* in the East China Sea is mainly limited to the Kuroshio and adjacent waters in the winter. In the summer, it could even expand to the Yangtze River estuary, covering most of the sea area (Jiao and Yang, 2002).

## 5 Conclusion

A “Nanfeng” cruise was conducted to explore the distribution, structure, and assembly patterns of eukaryotic and prokaryotic phytoplankton community in summer from PRE to the adjacent waters of Xisha Islands, the NSCS. Phytoplankton showed high biodiversity in the study area, with 7,181 ASVs detected for Dinophyta and 722 for Cyanophyta. Syndiniales represented the most abundant categorized order in eukaryotic phytoplankton, while *Prochlorococcus* was the most abundant genus in cyanobacteria. The three indices of Shannon-Wiener diversity, Pielou’s evenness, and Margalef richness showed the lowest values in the PRE area and gradually decreased with the depth, but cyanobacteria had higher values of alpha indices in the PRE and in the depths between 75 m and 750 m. The results of PCoA revealed significant differences in eukaryotic phytoplankton between various locations and depths, but no obvious differences were found in cyanobacteria communities between different areas. The morphological results differed from the data obtained through HTS. Specifically, diatoms (comprising 37 species) were found to dominate the phytoplankton community, with an average abundance of 3.01 × 10^4^ cells L^−1^. However, only six species of dinoflagellates were observed, and their density ranged from 0–2.86 × 10^3^ cells L^−1^. The distribution and structure of eukaryotic and prokaryotic phytoplankton were largely influenced by geographical location and environmental factors. The NCM and null model revealed that the deterministic processes were more significant than the stochastic processes in shaping the assembly of eukaryotic phytoplankton. Heterogeneous selection and homogeneous selection accounted for 47.27 and 29.95%, respectively. Conversely, the stochastic processes (over 60%) dominated the assembly of cyanobacteria, with the undominated processes accounting for 63.44%.

## Data availability statement

The datasets presented in this study can be found in online repositories. The names of the repository/repositories and accession number(s) can be found in the article/Supplementary material.

## Author contributions

JZ: Funding acquisition, Investigation, Software, Writing – original draft. YX: Visualization, Writing – original draft. PW: Methodology, Software, Writing – original draft. TW: Investigation, Software, Writing – original draft. LL: Investigation, Visualization, Writing – original draft. YuL: Investigation, Methodology, Writing – original draft. YoL: Funding acquisition, Supervision, Writing – review & editing. CL: Conceptualization, Formal analysis, Supervision, Writing – review & editing.

## References

[ref1] Anderson S. R. Harvey E. L. (2020). Temporal variability and ecological interactions of parasitic marine Syndiniales in coastal protist communities. mSphere 5, e00209–e00220. doi: 10.1128/mSphere.00209-20 32461270 PMC7253595

[ref2] Andersson A. Höglander H. Karlsson C. Huseby S. (2015). Key role of phosphorus and nitrogen in regulating cyanobacterial community composition in the northern Baltic Sea. Estuar. Coast. Shelf Sci. 164, 161–171. doi: 10.1016/j.ecss.2015.07.013

[ref3] Bendschneider K. Robinson R. J. (1952). A new spectrophotometric method for the determination of nitrite in sea water. J. Mar. Res. 11, 87–96.

[ref4] Bertos-Fortis M. Farnelid H. M. Lindh M. V. Casini M. Andersson A. Pinhassi J.. (2016). Unscrambling Cyanobacteria community dynamics related to environmental factors. Front. Microbiol. 7:625. doi: 10.3389/fmicb.2016.00625 27242679 PMC4860504

[ref5] Biller S. J. Berube P. M. Lindell D. Chisholm S. W. (2015). *Prochlorococcus*: the structure and function of collective diversity. Nat. Rev. Microbiol. 13, 13–27. doi: 10.1038/nrmicro3378, PMID: 25435307

[ref6] Burkholder J. M. Glibert P. M. Skelton H. M. (2008). Mixotrophy, a major mode of nutrition for harmful algal species in eutrophic waters. Harmful Algae 8, 77–93. doi: 10.1016/j.hal.2008.08.010

[ref7] Chen W. Ren K. Isabwe A. Chen H. Liu M. Yang J. (2019). Stochastic processes shape microeukaryotic community assembly in a subtropical river across wet and dry seasons. Microbiome 7:138. doi: 10.1186/s40168-019-0749-8, PMID: 31640783 PMC6806580

[ref8] Clarke L. J. Bestley S. Bissett A. Deagle B. E. (2019). A globally distributed Syndiniales parasite dominates the Southern Ocean micro-eukaryote community near the sea-ice edge. ISME J. 13, 734–737. doi: 10.1038/s41396-018-0306-7, PMID: 30367123 PMC6461979

[ref9] Cui L. Lu X. Dong Y. Cen J. Cao R. Pan L.. (2018). Relationship between phytoplankton community succession and environmental parameters in Qinhuangdao coastal areas, China: a region with recurrent brown tide outbreaks. Ecotoxicol. Environ. Saf. 159, 85–93. doi: 10.1016/j.ecoenv.2018.04.043, PMID: 29730413

[ref10] De Vargas C. Audic S. Henry N. Decelle J. Mahé F. Logares R.. (2015). Eukaryotic plankton diversity in the sunlit ocean. Science 348:1261605. doi: 10.1126/science.1261605, PMID: 25999516

[ref11] Diao C. Wang M. Zhong Z. Li Y. Xian W. Zhang H. (2023). Biodiversity exploration of Formosa ridge cold seep in the South China Sea using an eDNA metabarcoding approach. Mar. Environ. Res. 190:106109. doi: 10.1016/j.marenvres.2023.106109, PMID: 37506653

[ref12] Dini-Andreote F. Stegen J. C. Van Elsas J. D. Salles J. F. O. (2015). Disentangling mechanisms that mediate the balance between stochastic and deterministic processes in microbial succession. Proc. Natl. Acad. Sci. 112, 1326–1332. doi: 10.1073/pnas.1414261112 PMC4371938 25733885

[ref13] Dufrêne M. Legendre P. (1997). Species assemblages and indicator species: the need for a flexible asymmetrical approach. Ecol. Monogr. 67, 345–366. doi: 10.2307/2963459

[ref14] Ebina J. Tsutsui T. Shirai T. (1983). Simultaneous determination of total nitrogen and total phosphorus in water using peroxodisulfate oxidation. Water Res. 17, 1721–1726. doi: 10.1016/0043-1354(83)90192-6

[ref15] Edgar R. C. (2010). Search and clustering orders of magnitude faster than BLAST. Bioinformatics 26, 2460–2461. doi: 10.1093/bioinformatics/btq461, PMID: 20709691

[ref16] Edgar R. C. (2013). UPARSE: highly accurate OTU sequences from microbial amplicon reads. Nat. Methods 10, 996–998. doi: 10.1038/nmeth.2604, PMID: 23955772

[ref17] Field C. B. Behrenfeld M. J. Randerson J. T. Falkowski P. G. (1998). Primary production of the biosphere: integrating terrestrial and oceanic components. Science 281, 237–240. doi: 10.1126/science.281.5374.237, PMID: 9657713

[ref18] Gobler C. J. (2020). Climate change and harmful algal blooms: insights and perspective. Harmful Algae 91:101731. doi: 10.1016/j.hal.2019.101731 32057341

[ref19] Gong F. Li G. Wang Y. Liu Q. Huang F. Yin K.. (2020). Spatial shifts in size structure, phylogenetic diversity, community composition and abundance of small eukaryotic plankton in a coastal upwelling area of the northern South China Sea. J. Plankton Res. 42, 650–667. doi: 10.1093/plankt/fbaa046

[ref20] Guo Y. Zhang A. Qin C. Yu G. Ma H. (2023). Community assembly patterns and processes of microbiome responses to habitats and *Mytilopsis sallei* invasion in the tidal zones of the Pearl River estuary. Sci. Total Environ. 857:159675. doi: 10.1016/j.scitotenv.2022.159675 36280051

[ref21] Hallegraeff G. M. (2010). Ocean climate change, phytoplankton community responses, and harmful algal blooms: a formidable predictive challenge. J. Phycol. 46, 220–235. doi: 10.1111/j.1529-8817.2010.00815.x

[ref22] Ho T. Pan X. Yang H. Wong G. T. F. Shiah F. (2015). Controls on temporal and spatial variations of phytoplankton pigment distribution in the northern South China Sea. Deep-Sea Res. II Top. Stud. Oceanogr. 117, 65–85. doi: 10.1016/j.dsr2.2015.05.015

[ref23] Huang X. P. Huang L. M. Yue W. Z. (2003). The characteristics of nutrients and eutrophication in the Pearl River estuary, South China. Mar. Pollut. Bull. 47, 30–36. doi: 10.1016/S0025-326X(02)00474-5, PMID: 12787594

[ref24] Huang C. Zeng T. Ye F. Xie L. Wang Z. Wei G.. (2018). Natural and anthropogenic impacts on environmental changes over the past 7500 years based on the multi-proxy study of shelf sediments in the northern South China Sea. Quat. Sci. Rev. 197, 35–48. doi: 10.1016/j.quascirev.2018.08.005

[ref25] Jäger C. G. Diehl S. Schmidt G. M. (2008). Influence of water-column depth and mixing on phytoplankton biomass, community composition, and nutrients. Limnol. Oceanogr. 53, 2361–2373. doi: 10.4319/lo.2008.53.6.2361

[ref26] Jiao N. Yang Y. (2002). Ecological studies on *Prochlorococcus* in China seas. Chin. Sci. Bull. 47, 1243–1250. doi: 10.1360/02tb9276

[ref27] Jiao S. Yang Y. Xu Y. Zhang J. Lu Y. (2020). Balance between community assembly processes mediates species coexistence in agricultural soil microbiomes across eastern China. ISME J. 14, 202–216. doi: 10.1038/s41396-019-0522-9, PMID: 31611655 PMC6908645

[ref28] Kang J. Mohamed H. F. Liu X. Pei L. Huang S. Lin X.. (2022). Combined culture and DNA metabarcoding analysis of cyanobacterial community structure in response to coral reef health status in the South China Sea. J. Mar. Sci. Eng. 10:1984. doi: 10.3390/jmse10121984

[ref29] Klais R. Norros V. Lehtinen S. Tamminen T. Olli K. (2017). Community assembly and drivers of phytoplankton functional structure. Funct. Ecol. 31, 760–767. doi: 10.1111/1365-2435.12784

[ref30] Levin L. A. (2005). Ecology of cold seep sediments: interactions of fauna with flow, chemistry and microbes. Oceanogr. Mar. Biol. 20051650, 1–46. doi: 10.1201/9781420037449.ch1

[ref31] Li L. Lü S. Cen J. (2019). Spatio-temporal variations of harmful algal blooms along the coast of Guangdong, southern China during 1980–2016. J. Oceanol. Limnol. 37, 535–551. doi: 10.1007/s00343-019-8088-y

[ref32] Li X. Warren A. Jiao N. Xu D. (2020). Distribution of protists in the deep South China Sea revealed by high-throughput sequencing. J. Ocean Univ. China 19, 161–170. doi: 10.1007/s11802-020-4137-6

[ref33] Lu S. Ou L. Dai X. Cui L. Dong Y. Wang P.. (2022). An overview of *Prorocentrum donghaiense* blooms in China: species identification, occurrences, ecological consequences, and factors regulating prevalence. Harmful Algae 114:102207. doi: 10.1016/j.hal.2022.102207, PMID: 35550289

[ref34] Monchamp M.-E. Spaak P. Domaizon I. Dubois N. Bouffard D. Pomati F. (2018). Homogenization of lake cyanobacterial communities over a century of climate change and eutrophication. Nat. Ecol. Evol. 2, 317–324. doi: 10.1038/s41559-017-0407-0, PMID: 29230026

[ref35] Mora D. Abarca N. Proft S. Grau J. H. Enke N. Carmona J.. (2019). Morphology and metabarcoding: a test with stream diatoms from Mexico highlights the complementarity of identification methods. Freshw. Sci. 38, 448–464. doi: 10.1086/704827

[ref36] Mullin J. B. Riley J. (1955). The spectrophotometric determination of nitrate in natural waters, with particular reference to sea-water. Anal. Chim. Acta 12, 464–480. doi: 10.1016/S0003-2670(00)87865-4

[ref37] Murphy J. Riley J. P. (1962). A modified single solution method for the determination of phosphate in natural waters. Anal. Chim. Acta 27, 31–36. doi: 10.1016/S0003-2670(00)88444-5

[ref38] Peng C. Wang K. Wang W. Kuang F. Gao Y. Jiang R.. (2023). Phytoplankton community structure and environmental factors during the outbreak of crown-of-thorns starfish in Xisha Islands, South China Sea. Environ. Res. 235:116568. doi: 10.1016/j.envres.2023.116568 37422114

[ref39] Pile A. J. Young C. M. (1999). Plankton availability and retention efficiencies of cold-seep symbiotic mussels. Limnol. Oceanogr. 44, 1833–1839. doi: 10.4319/lo.1999.44.7.1833

[ref40] Rosindell J. Hubbell S. P. Etienne R. S. (2011). The unified neutral theory of biodiversity and biogeography at age ten. Trends Ecol. Evol. 26, 340–348. doi: 10.1016/j.tree.2011.03.024, PMID: 21561679

[ref41] Sagi T. (1966). Determination of ammonia in sea water by the indophenol method and its application to the coastal and off-shore waters. Oceanogr. Mag. 18, 43–51.

[ref42] Sloan W. T. Lunn M. Woodcock S. Head I. M. Nee S. Curtis T. P. (2006). Quantifying the roles of immigration and chance in shaping prokaryote community structure. Environ. Microbiol. 8, 732–740. doi: 10.1111/j.1462-2920.2005.00956.x, PMID: 16584484

[ref43] Stegen J. C. Lin X. Fredrickson J. K. Chen X. Kennedy D. W. Murray C. J.. (2013). Quantifying community assembly processes and identifying features that impose them. ISME J. 7, 2069–2079. doi: 10.1038/ismej.2013.93, PMID: 23739053 PMC3806266

[ref44] Stoeck T. Hayward B. Taylor G. T. Varela R. Epstein S. S. (2006). A multiple PCR-primer approach to access the microeukaryotic diversity in environmental samples. Protist 157, 31–43. doi: 10.1016/j.protis.2005.10.004, PMID: 16431157

[ref45] Utermohl H. (1958). Zur Vervollkommung der quantitativen phytoplankton-methodik. Verhandlungen des Internationalen Verein Limnologie 9:38.

[ref46] Wang Z. Akbar S. Sun Y. Gu L. Zhang L. Lyu K.. (2021). Cyanobacterial dominance and succession: factors, mechanisms, predictions, and managements. J. Environ. Manag. 297:113281. doi: 10.1016/j.jenvman.2021.113281, PMID: 34274765

[ref47] Wang Z. Liu L. Tang Y. Li A. Liu C. Xie C.. (2022). Phytoplankton community and HAB species in the South China Sea detected by morphological and metabarcoding approaches. Harmful Algae 118:102297. doi: 10.1016/j.hal.2022.102297, PMID: 36195422

[ref48] Wasmund N. Nausch G. Voss M. (2012). Upwelling events may cause Cyanobacteria blooms in the Baltic Sea. J. Mar. Syst. 90, 67–76. doi: 10.1016/j.jmarsys.2011.09.001

[ref49] Wei N. Satheeswaran T. Jenkinson I. R. Xue B. Wei Y. Liu H.. (2018). Factors driving the spatiotemporal variability in phytoplankton in the northern South China Sea. Cont. Shelf Res. 162, 48–55. doi: 10.1016/j.csr.2018.04.009

[ref50] Wu W. Lu H.-P. Sastri A. Yeh Y.-C. Gong G.-C. Chou W.-C.. (2018). Contrasting the relative importance of species sorting and dispersal limitation in shaping marine bacterial versus protist communities. ISME J. 12, 485–494. doi: 10.1038/ismej.2017.183, PMID: 29125596 PMC5776463

[ref51] Xiao W. Liu X. Irwin A. J. Laws E. A. Wang L. Chen B.. (2018). Warming and eutrophication combine to restructure diatoms and dinoflagellates. Water Res. 128, 206–216. doi: 10.1016/j.watres.2017.10.051, PMID: 29107905

[ref52] Xu Z. Chen Y. (1989). Aggregated intensity of dominant species of zooplankton in autumn in the East China Sea and Yellow Sea. Chinese J. Ecol. 8, 13–15.

[ref53] Xu Z. Cheung S. Endo H. Xia X. Wu W. Chen B.. (2022). Disentangling the ecological processes shaping the latitudinal pattern of phytoplankton communities in the Pacific Ocean. mSystems 7, e01203–e01221. doi: 10.1128/msystems.01203-21 35089068 PMC8725599

[ref54] Xu S. Xiao Y. Xu Y. Su L. Cai Y. Qi Z.. (2024). Effects of seasonal variations and environmental factors on phytoplankton community structure and abundance in Beibu gulf, China. Ocean Coast. Manag. 248:106982. doi: 10.1016/j.ocecoaman.2023.106982

[ref55] Zhang W. Han H. Qiu L. Liu C. Zhang Q. Zhou G. (2022). Variations in nano- and pico-eukaryotic phytoplankton assemblages in the Qinhuangdao green-tide area. J. Oceanol. Limnol. 40, 2446–2461. doi: 10.1007/s00343-022-2198-7

[ref56] Zhang Y. Huang N. Wang M. Liu H. Jing H. (2021). Microbial eukaryotes associated with sediments in deep-sea cold seeps. Front. Microbiol. 12:782004. doi: 10.3389/fmicb.2021.782004, PMID: 35003010 PMC8740301

[ref57] Zhang S. Zeng Y. Zhu J. Cai Z. Zhou J. (2022). The structure and assembly mechanisms of plastisphere microbial community in natural marine environment. J. Hazard. Mater. 421:126780. doi: 10.1016/j.jhazmat.2021.126780, PMID: 34358974

[ref58] Zhao L. Chen R. Lou L. Jing X. Liu Q. Liu J.. (2020). Layer-by-layer-assembled antifouling films with surface microtopography inspired by *Laminaria japonica*. Appl. Surf. Sci. 511:145564. doi: 10.1016/j.apsusc.2020.145564

[ref59] Zhao Y. Xu T. Law Y. S. Feng D. Qiu J. W. (2020). Ecological characterization of cold-seep epifauna in the South China Sea. Deep Sea Res. I Oceanogr. Res. Papers 163:103361. doi: 10.1016/j.dsr.2020.103361

[ref60] Zhou J. Ning D. (2017). Stochastic community assembly: does it matter in microbial ecology? Microbiol. Mol. Biol. Rev. 81:e00002-17. doi: 10.1128/MMBR.00002-17, PMID: 29021219 PMC5706748

[ref61] Zou J. Xie H. Zheng C. Songhui L. (2022). Spatial-temporal distribution of *Prorocentrum concavum* population in relation to environmental factors in Xincun Bay, a tropical coastal lagoon in China. Front. Mar. Sci. 9:931533. doi: 10.3389/fmars.2022.931533

