# Supplementary material for: Distribution, community structure and assembly patterns of phytoplankton in the northern South China Sea
Source: Front Microbiol. 2024 Jul 31;15:1450706. doi: 10.3389/fmicb.2024.1450706 (PMC11322478; doi:10.3389/fmicb.2024.1450706)
Supplement: Supplementary file 1 [file Data_Sheet_1.DOCX]

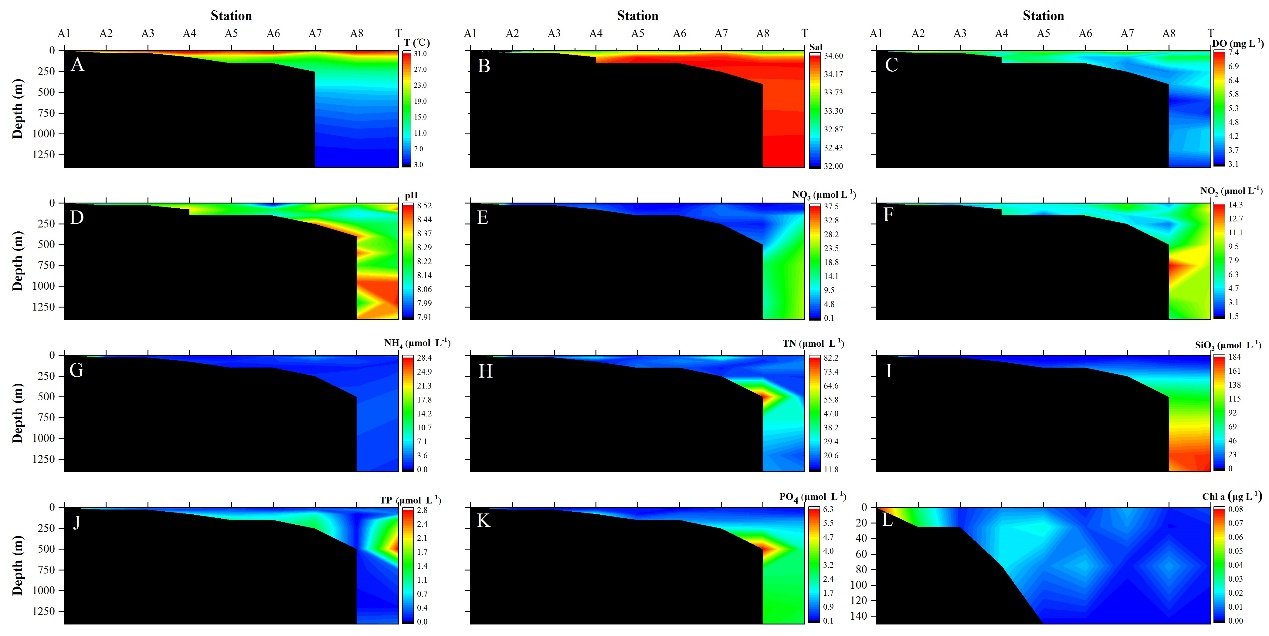


**Fig. S1** The distribution of environmental factors in northern South China Sea during August 2023


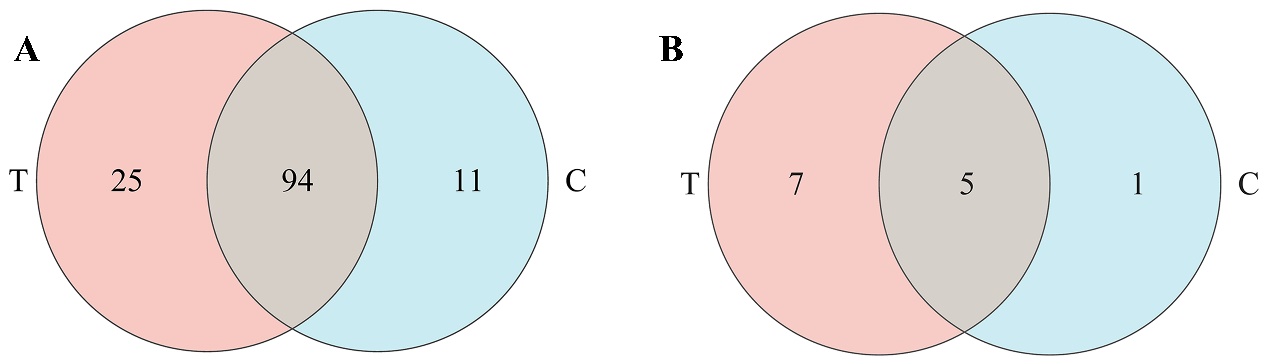


**Fig. S2** Venn analysis showing the specific ASVs of eukaryotic (A) and prokaryotic (B) phytoplankton between station T and station C


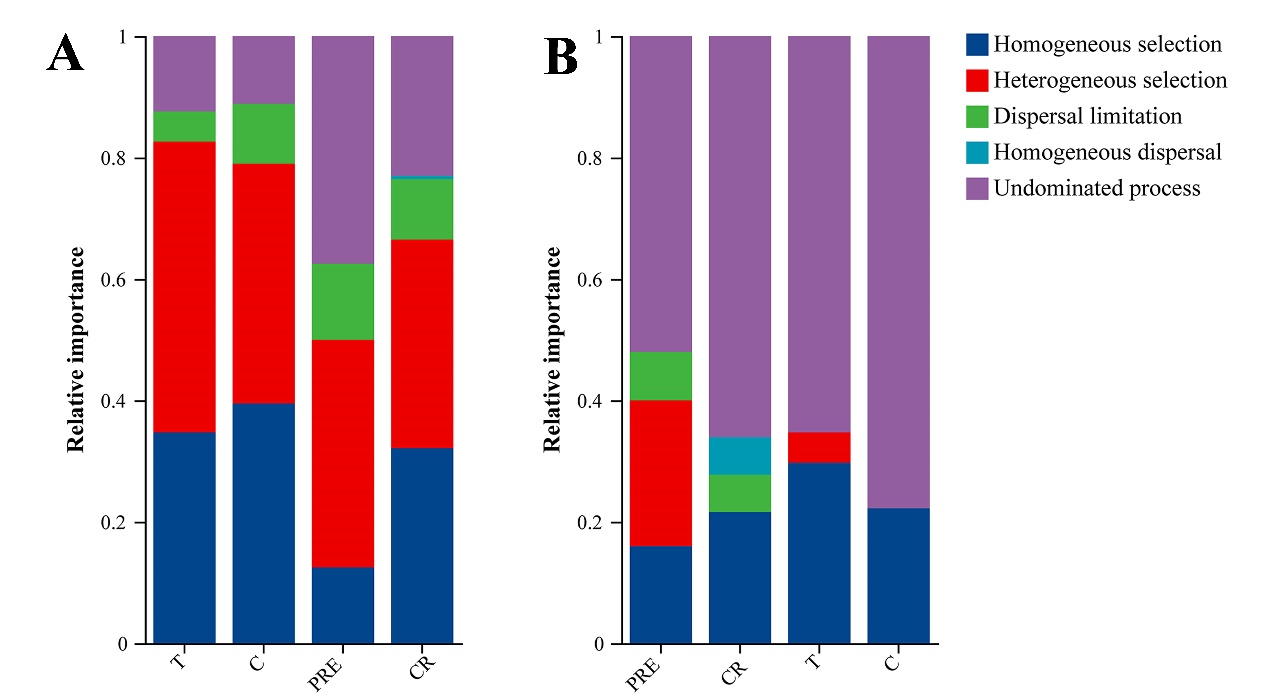


**Fig. S3** Null model showing the contributions of different ecological processes to the assembly of phytoplankton in different areas of the northern South China Sea during August 2023. PRE, Pearl River Estuary; CR, central area; C, Site C; T, Site T

**Table S1** Coefficients of Spearman correlations between environmental and eukaryotic alpha diversity indices. The values without underline indicate the r^2^, and the values with underline indicate significant level. P<0.05 was showed bold font. *D, J’*, and *H’* indicate Margalef richness index, Pielou’s evenness index and Shannon-Wiener diversity index, respectively

| Sample | Lon | Lat | Depth | T | Sal | DO | pH | Chl a | NO_3_ | NO_2_ | NH_4_ | TN | SiO_3_ | PO_4_ | TP | *D* | *J'* | *H'* |
| --- | --- | --- | --- | --- | --- | --- | --- | --- | --- | --- | --- | --- | --- | --- | --- | --- | --- | --- |
| Lon |  | **0.00** | 0.07 | 0.27 | **0.04** | 0.35 | 0.85 | **0.00** | 0.22 | **0.00** | 0.11 | 0.11 | 0.28 | 0.69 | 0.64 | **0.01** | **0.04** | **0.01** |
| Lat | 1.00 |  | 0.06 | 0.24 | **0.01** | 0.40 | 0.99 | **0.00** | 0.10 | **0.00** | **0.05** | 0.05 | 0.17 | 0.57 | 0.56 | **0.01** | **0.02** | **0.00** |
| Depth | -0.34 | -0.34 |  | **0.00** | 0.11 | **0.00** | 0.43 | **0.04** | 1.00 | 0.43 | 0.21 | 0.37 | 0.19 | **0.00** | **0.00** | 0.93 | 0.41 | 0.68 |
| T | 0.18 | 0.18 | -0.92 |  | 0.09 | **0.00** | 0.77 | 0.17 | 0.99 | 0.31 | 0.30 | 0.43 | 0.21 | **0.00** | **0.00** | 0.72 | 0.45 | 0.91 |
| Sal | -0.26 | -0.26 | 0.93 | -0.93 |  | 0.48 | 0.70 | **0.00** | **0.00** | 0.06 | **0.00** | **0.00** | **0.00** | 0.05 | 0.16 | 0.07 | **0.00** | **0.00** |
| DO | 0.13 | 0.13 | -0.61 | 0.54 | -0.52 |  | 0.43 | 0.91 | **0.04** | 0.56 | 0.19 | 0.16 | **0.01** | **0.00** | **0.03** | 0.17 | 0.70 | 0.47 |
| pH | 0.01 | 0.01 | -0.12 | 0.15 | -0.05 | -0.13 |  | 0.55 | 0.53 | 0.55 | 0.45 | 0.24 | 0.94 | 0.85 | 0.89 | 0.05 | 0.64 | 0.13 |
| Chl a | 0.58 | 0.58 | -0.50 | 0.33 | -0.42 | 0.23 | 0.19 |  | **0.00** | 0.11 | **0.00** | **0.00** | **0.00** | 0.11 | 0.38 | **0.02** | **0.00** | **0.00** |
| NO_3_ | -0.08 | -0.08 | 0.43 | -0.48 | 0.45 | -0.55 | 0.21 | -0.19 |  | 0.36 | **0.00** | **0.00** | **0.00** | **0.00** | **0.01** | 0.13 | **0.00** | **0.00** |
| NO_2_ | -0.50 | -0.50 | 0.23 | -0.19 | 0.24 | -0.06 | 0.06 | -0.17 | 0.24 |  | 0.19 | 0.14 | 0.15 | 0.58 | 0.85 | 0.12 | 0.07 | **0.05** |
| NH_4_ | -0.06 | -0.06 | -0.19 | 0.09 | -0.05 | -0.12 | 0.21 | 0.26 | 0.24 | -0.01 |  | **0.00** | **0.00** | **0.02** | 0.15 | 0.12 | **0.00** | **0.00** |
| TN | 0.14 | 0.14 | 0.06 | -0.07 | 0.05 | -0.20 | 0.29 | 0.03 | 0.31 | -0.22 | 0.18 |  | **0.00** | 0.08 | 0.23 | 0.36 | **0.00** | 0.01 |
| SiO_3_ | -0.23 | -0.23 | 0.66 | -0.74 | 0.64 | -0.58 | -0.05 | -0.28 | 0.59 | 0.05 | 0.17 | 0.08 |  | **0.00** | **0.00** | **0.07** | **0.00** | **0.00** |
| PO_4_ | -0.07 | -0.07 | 0.51 | -0.57 | 0.51 | -0.54 | -0.02 | -0.22 | 0.62 | 0.02 | 0.07 | 0.00 | 0.82 |  | **0.00** | 0.19 | **0.03** | 0.06 |
| TP | 0.16 | 0.16 | 0.36 | -0.50 | 0.34 | -0.44 | -0.03 | -0.10 | 0.34 | -0.02 | -0.09 | -0.03 | 0.57 | 0.48 |  | 0.13 | 0.21 | 0.12 |
| *D* | -0.39 | -0.39 | 0.11 | 0.07 | 0.04 | -0.19 | 0.44 | -0.24 | 0.01 | 0.28 | -0.05 | 0.12 | -0.21 | -0.25 | -0.29 |  | **0.01** | **0.00** |
| *J'* | -0.13 | -0.13 | 0.21 | -0.12 | 0.17 | -0.12 | 0.28 | -0.14 | -0.15 | 0.34 | -0.31 | -0.06 | -0.13 | -0.06 | 0.17 | 0.32 |  | **0.00** |
| *H'* | -0.34 | -0.34 | 0.05 | 0.14 | -0.01 | -0.15 | 0.49 | -0.12 | -0.08 | 0.33 | -0.05 | 0.05 | -0.30 | -0.32 | -0.22 | 0.94 | 0.52 |  |

**Table S2** Coefficients of Spearman correlations between environmental and Cyanobacteria diversity indices. The values without underline indicate the r^2^, and the values with underline indicate significant level. P<0.05 was showed bold font. *D, J’*, and *H’* indicate Margalef richness index, Pielou’s evenness index and Shannon-Wiener diversity index, respectively

| Sample | Lon | Lat | Depth | T | Sal | DO | pH | Chl a | NO_3_ | NO_2_ | NH_4_ | TN | SiO_3_ | PO_4_ | TP | *D* | *J'* | *H'* |
| --- | --- | --- | --- | --- | --- | --- | --- | --- | --- | --- | --- | --- | --- | --- | --- | --- | --- | --- |
| Lon |  | **0.00** | 0.07 | 0.27 | **0.04** | 0.35 | 0.85 | **0.00** | 0.22 | **0.00** | 0.11 | 0.11 | 0.28 | 0.69 | 0.64 | **0.01** | **0.02** | **0.01** |
| Lat | 1.00 |  | 0.06 | 0.24 | **0.01** | 0.40 | 0.99 | **0.00** | 0.10 | **0.00** | 0.05 | 0.05 | 0.17 | 0.57 | 0.56 | **0.00** | **0.01** | **0.00** |
| Depth | -0.34 | -0.34 |  | **0.00** | 0.11 | **0.00** | 0.43 | **0.04** | 1.00 | 0.43 | 0.21 | 0.37 | 0.19 | **0.00** | **0.00** | 0.65 | 0.67 | 0.61 |
| T | 0.18 | 0.18 | -0.92 |  | 0.09 | **0.00** | 0.77 | 0.17 | 0.99 | 0.31 | 0.30 | 0.43 | 0.21 | **0.00** | **0.00** | 0.94 | 0.81 | 0.83 |
| Sal | -0.26 | -0.26 | 0.93 | -0.93 |  | 0.48 | 0.70 | **0.00** | **0.00** | 0.06 | **0.00** | **0.00** | **0.00** | 0.05 | 0.16 | **0.01** | **0.00** | **0.00** |
| DO | 0.13 | 0.13 | -0.61 | 0.54 | -0.52 |  | 0.43 | 0.91 | **0.04** | 0.56 | 0.19 | 0.16 | **0.01** | **0.00** | **0.03** | 0.12 | 0.79 | 0.52 |
| pH | 0.01 | 0.01 | -0.12 | 0.15 | -0.05 |  |  | 0.55 | 0.53 | 0.55 | 0.45 | 0.24 | 0.94 | 0.85 | 0.89 | 0.09 | 0.36 | 0.18 |
| Chl a | 0.58 | 0.58 | -0.50 | 0.33 | -0.42 | 0.23 | 0.19 |  | **0.00** | 0.11 | **0.00** | **0.00** | **0.00** | 0.11 | 0.38 | **0.00** | **0.00** | **0.00** |
| NO_3_ | -0.08 | -0.08 | 0.43 | -0.48 | 0.45 | -0.55 | 0.21 | -0.19 |  | 0.36 | **0.00** | **0.00** | **0.00** | **0.00** | **0.01** | 0.05 | **0.00** | **0.00** |
| NO_2_ | -0.50 | -0.50 | 0.23 | -0.19 | 0.24 | -0.06 | 0.06 | -0.17 | 0.24 |  | 0.19 | 0.14 | 0.15 | 0.58 | 0.85 | 0.09 | 0.07 | 0.05 |
| NH_4_ | -0.06 | -0.06 | -0.19 | 0.09 | -0.05 | -0.12 | 0.21 | 0.26 | 0.24 | -0.01 |  | **0.00** | **0.00** | **0.02** | 0.15 | **0.03** | **0.00** | **0.00** |
| TN | 0.14 | 0.14 | 0.06 | -0.07 | 0.05 | -0.20 | 0.29 | 0.03 | 0.31 | -0.22 | 0.18 |  | **0.00** | 0.08 | 0.23 | 0.18 | **0.00** | **0.00** |
| SiO_3_ | -0.23 | -0.23 | 0.66 | -0.74 | 0.64 | -0.58 | -0.05 | -0.28 | 0.59 | 0.05 | 0.17 | 0.08 |  | **0.00** | **0.00** | **0.03** | **0.00** | **0.00** |
| PO_4_ | -0.07 | -0.07 | 0.51 | -0.57 | 0.51 | -0.54 | -0.02 | -0.22 | 0.62 | 0.02 | 0.07 | 0.00 | 0.82 |  | **0.00** | 0.18 | **0.02** | 0.06 |
| TP | 0.16 | 0.16 | 0.36 | -0.50 | 0.34 | -0.44 | -0.03 | -0.10 | 0.34 | -0.02 | -0.09 | -0.03 | 0.57 | 0.48 |  | 0.17 | 0.13 | 0.13 |
| *D* | -0.35 | -0.35 | 0.16 | 0.03 | 0.07 | -0.21 | 0.39 | -0.19 | -0.06 | 0.28 | -0.10 | 0.09 | -0.22 | -0.28 | -0.26 |  | **0.00** | **0.00** |
| *J'* | -0.17 | -0.17 | -0.03 | 0.18 | -0.06 | -0.03 | 0.54 | -0.07 | -0.12 | 0.34 | -0.18 | -0.06 | -0.37 | -0.26 | -0.10 | 0.75 |  | **0.00** |
| *H'* | -0.31 | -0.31 | 0.06 | 0.12 | -0.01 | -0.16 | 0.46 | -0.11 | -0.12 | 0.30 | -0.08 | 0.02 | -0.31 | -0.32 | -0.19 | 0.96 | 0.87 |  |

**Table S3** Coefficient of correlation between environmental factors and eukaryotic phytoplankton on the genus level of the first two axes from redundancy analysis. The bold values (p<0.05) indicated the significant correlations

| **Environmental factors** | **RDA1** | **RDA2** | **r^2^** | ***p*** |
| --- | --- | --- | --- | --- |
| Longitude | -0.9603 | 0.2789 | 0.2083 | **0.019** |
| Latitude | -0.9315 | 0.3636 | 0.2553 | **0.01** |
| Depth | 0.9256 | 0.3785 | 0.7492 | **0.001** |
| T | -0.9313 | -0.3642 | 0.6293 | **0.001** |
| Sal | 0.6632 | -0.7484 | 0.9816 | **0.001** |
| DO | -0.4608 | -0.8875 | 0.3382 | **0.002** |
| pH | -0.988 | -0.1547 | 0.0461 | 0.548 |
| Chl a | -0.7428 | 0.6695 | 0.7146 | **0.002** |
| NO_3_ | -0.4643 | 0.8857 | 0.9246 | **0.002** |
| NO_2_ | 0.6256 | -0.7802 | 0.0892 | 0.283 |
| NH_4_ | -0.6138 | 0.7895 | 0.9515 | **0.019** |
| TN | -0.5982 | 0.8013 | 0.7824 | **0.018** |
| SiO_3_ | -0.286 | 0.9582 | 0.8799 | **0.001** |
| PO_4_ | 0.2244 | 0.9745 | 0.4645 | **0.002** |
| TP | 0.2725 | 0.9622 | 0.2653 | **0.047** |

**Table S4** Coefficient of correlation between environmental factors and cyanobacteria of the first two axes from redundancy analysis. The bold values (p<0.05) indicated the significant correlations

| **Environmental factors** | **RDA1** | **RDA2** | **r^2^** | ***p*** |
| --- | --- | --- | --- | --- |
| Longitude | -0.3487 | 0.9372 | 0.1921 | 0.054 |
| Latitude | -0.3336 | 0.9427 | 0.2531 | **0.021** |
| Depth | -0.6461 | -0.7632 | 0.6127 | **0.001** |
| T | 0.689 | 0.7247 | 0.7048 | **0.001** |
| Sal | 0.2121 | -0.9773 | 0.8679 | **0.001** |
| DO | 0.7837 | 0.6211 | 0.0962 | 0.232 |
| pH | 0.8756 | 0.483 | 0.0501 | 0.462 |
| Chl a | -0.2763 | 0.9611 | 0.6703 | **0.002** |
| NO_3_ | -0.3954 | 0.9185 | 0.6439 | **0.026** |
| NO_2_ | -0.0625 | -0.998 | 0.092 | 0.256 |
| NH_4_ | -0.2433 | 0.9699 | 0.7002 | **0.024** |
| TN | -0.2125 | 0.9772 | 0.6094 | **0.013** |
| SiO_3_ | -0.6086 | 0.7935 | 0.5658 | **0.019** |
| PO_4_ | -0.994 | 0.1094 | 0.3686 | **0.004** |
| TP | -0.9895 | 0.1447 | 0.3326 | **0.01** |
